# Physicochemical, genomic, and phenotypic characterization of *Escherichia* phage BME3

**DOI:** 10.1128/spectrum.01301-24

**Published:** 2025-05-22

**Authors:** Belén Toaquiza-Vilca, Diego Quito-Avila, Pedro Maldonado-Alvarado, Omar Ruiz-Barzola, Alexis Debut, Marynes Montiel

**Affiliations:** 1 Department of Food Science and Biotechnology, Escuela Politécnica Nacional (EPN) 27882, Quito, Ecuador; 2 Facultad de Ciencias de la Vida, Escuela Superior Politécnica del Litoral, ESPOL, Campus Gustavo Galindo 27883, Guayaquil, Ecuador; 3 Centro de Investigaciones Biotecnológicas del Ecuador, CIBE, Escuela Superior Politécnica del Litoral, ESPOL, Campus Gustavo Galindo, Guayaquil, Ecuador; 4 Departamento de Estadística, Universidad de Salamanca, Salamanca, Spain; 5 Centro de Nanociencia y Nanotecnología, Universidad de las Fuerzas Armadas ESPE, Sangolquí, Ecuador; University of Pittsburgh School of Medicine, Pittsburgh, Pennsylvania, USA

**Keywords:** bacteriophage, *Escherichia coli*, *Caudoviricetes*, *Justusliebigvirus*

## Abstract

Infections caused by pathogenic *Escherichia coli* strains are increasing, and with the rising of antimicrobial resistance among bacterial pathogens, alternative therapeutic options are being actively explored, including phage therapy. In this research, a new bacteriophage, provisionally named BME3, with lytic activity against *Escherichia coli* was identified and characterized at the physicochemical, morphological, and genetic levels. BME3 was isolated from the tropical estuarine waters of Estero Salado, Guayaquil, Ecuador. Subsequently, it was purified and amplified, followed by a series of tests that included host range, stability studies, morphological characterization by transmission electron microscopy (TEM), and whole genome sequencing. The genomic analysis revealed that BME3 is closely related to members of the genus *Justusliebigvirus*, with a double-stranded DNA genome of 147,371 bp in length, a GC content of 37.5%, and 16 tRNA genes. In addition, BME3 lacks genes associated with lysogenesis, antibiotic resistance, or virulence. BME3 infected approximately 48% (13/27) of environmental *E. coli* strains. Among these, the infection rate was higher for antibiotic-resistant strains (67%) compared to intermediate and sensitive strains (33%). The phage infected *E. coli* and *Salmonella* sp. strains but did not affect *Bacillus* sp., *Pseudomonas* sp., or *Vibrio* sp. Moreover, BME3 was found to be stable at temperatures below 60°C, in pH ranges between 5 and 9, and was not sensitive to chloroform. TEM analysis supported the genetic sequence that assigned BME3 to the class *Caudoviricetes*. Phenotypic, genomic, and physicochemical characterization suggests that BME3 represents a promising option for phage therapy, with the potential to control antibiotic-resistant bacteria.

**IMPORTANCE:**

Although metagenomics offers a wealth of information, not all microorganisms can be isolated and cultivated in the laboratory. In this study, we successfully isolated and characterized a phage belonging to the *Justusliebigvirus* genus. This group has been poorly studied regarding its physicochemical properties and lysis profile against antibiotic-resistant environmental bacteria. These bacteriophages have received less attention compared to well-studied models such as phage T4. The isolation and characterization of the indigenous polyvalent bacteriophage BME3, obtained from tropical estuarine waters in Ecuador, provide valuable insights into its potential applications for environmental control of *Escherichia coli* and for mitigating the spread of bacterial resistance.

## INTRODUCTION

Bacteriophages are widely distributed in the biosphere and are estimated to have an abundance of approximately 10^31^ viral particles (1). Bacteriophages are classified according to their life cycle as lysogenic (temperate) and lytic (virulent). The former favors transduction, the horizontal transfer of antibiotic-resistance genes or toxins between bacteria, while the latter causes host lysis, a desired characteristic in phage therapy (2).

The isolation of bacteriophages from environmental samples has shown a greater abundance and diversity of dsDNA phages belonging to the class *Caudoviricetes*, commonly known as tailed bacteriophages (3). The presence of tailed bacteriophages in environmental samples shows a higher prevalence of the *Myoviridae* morphotype, followed by *Siphoviridae* and *Podoviridae* (4, 5). According to the taxonomic classification proposed by the International Committee on Taxonomy of Viruses (6), these morphotypes are represented by families such as *Drexlerviridae*, *Demerecviridae*, *Straboviridae*, *Chaseviridae*, and *Autographiviridae*.

Furthermore, the classification includes subfamilies such as *Ounavirinae*, *Vequintavirinae*, *Stephanstirmvirinae*, *Guernseyvirinae*, *Queuovirinae*, and *Gordonclarkvirinae*, as well as notable genera like *Dhillonvirus*, *Murrayvirus*, *Skarprettervirus*, and *Sortsnevirus* (4, 5, 7, 8).

The subfamily *Stephanstirmvirinae* comprises two genera: *Justusliebigvirus* and *Phapecoctavirus*, which include large enterobacteria-infecting myoviruses with genomes typically ranging from 146 to 149 kbp and a GC content of ~35% (4, 5). Within the genus *Justusliebigvirus*, species such as alia, muut, PD06, PHB05, phi92, and VecB have been reported (6), with phi92 being the most studied species of this genus (9). In 2020, Olsen et al. (4) isolated coliphages from water using the *Escherichia coli* strain MG1655, K-12, and identified a total of 136 phages, of which 5 belonged to the genus *Justusliebigvirus* (10). An additional characteristic of members of this genus is their wide host range that includes encapsulated and non-encapsulated enterobacteria (11, 12). This feature is facilitated by tail fibers and spikes located on the baseplate, enabling the recognition of diverse receptors on bacterial surfaces.

Bacteria employ various defense strategies against phages, including restriction-modification systems, CRISPR-Cas, and abortive infection mechanisms (13). However, bacteriophages continuously coevolve to evade these defenses, resulting in a constant “arms race” between bacteria and phages. This evolutionary battle imposes significant energetic and metabolic costs on bacteria, as they must maintain multiple defense mechanisms (14).

In this study, we present the isolation and characterization of a lytic bacteriophage, designated BME3, which infects *Escherichia coli* ATCC 15597. This phage was found in a tropical environment and is genetically closest to the partially characterized *Escherichia* phage Paula. To our knowledge, this is the first complete characterization of an environmental phage in Ecuador. The characterization of phage is a crucial step toward phage therapy as a promising alternative to combat antibiotic resistance.

## RESULTS AND DISCUSSION

### Isolation and morphological characterization of bacteriophage BME3

**Fig 1:**
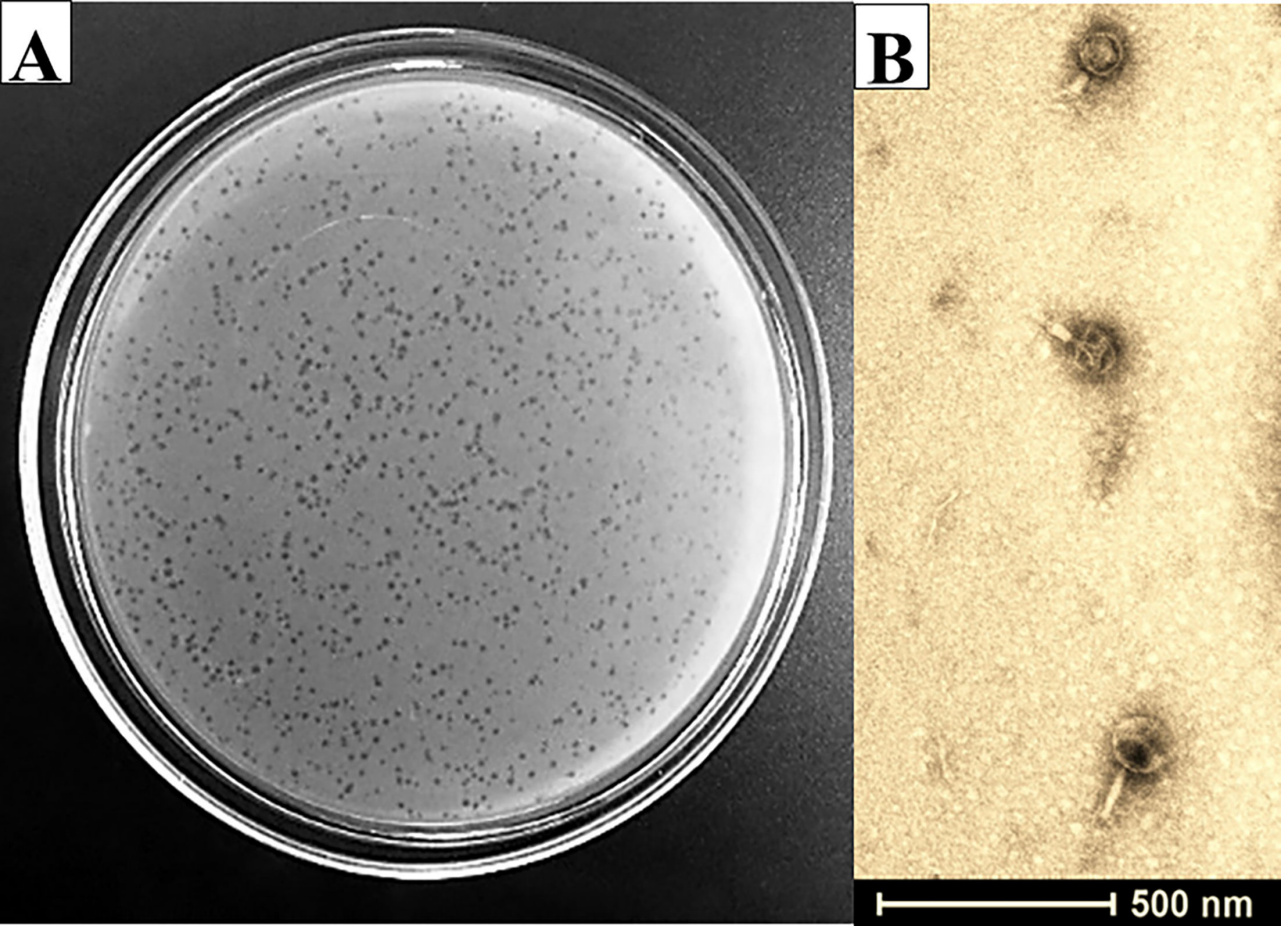
Characterization of bacteriophage BME3. (**A**) Plaque morphology of BME3 formed against *E. coli* ATCC 15597 determined by double-layer agar method. BME3 produces clear plaques with a diameter of less than 1 mm. (**B**) Transmission electron microscopy (TEM). Morphology of BME3 with a scale bar (200 nm). The phage virion was stained with 2% phosphotungstic acid and observed by TEM.

BME3 was isolated from estuarine water sample, with a coliphage count of 58 PFU/100 mL, collected from Estero Salado using *E. coli* strain ATCC 15597, which is known for its susceptibility to bacteriophages (15). Additionally, another coliphage belonging to the genus *Dhillonvirus* was identified in this sample. Species of this genus have been isolated from fecal samples of swine and cattle (16, 17). BME3 produced clear lytic plaques with a diameter of less than 1 mm (Fig. 1A). These small plaques may be attributed to the slow diffusion of large-headed bacteriophages (*Myoviridae*) through the top layer of agar (18).

Transmission electron microscopy (TEM) analysis of BME3 revealed morphological features similar to those typically observed in members of the *Myoviridae* within the *Caudoviricetes* (tailed phages) (10). The length of the icosahedral head and contractile tail was approximately 102.64 ± 11.57 nm and 105 ± 9 nm, respectively (Fig. 1B).

### Host range determination

The bacteriophage BME3 was selected for this study based on its lytic activity against various environmental *Escherichia coli* strains, compared to other bacteriophages recovered in the analyzed samples. BME3 exhibited a wide host range and could lyse strains of *E. coli* and *Salmonella* sp. but did not react against strains of *Bacillus* sp., *Pseudomonas* sp., and *Vibrio* sp. (Table 1).

**TABLE 1:** Activity of bacteriophage BME3 against environmental bacterial strains

Species	ID	Source	Antibiotic resistance^*a*^	Sensitivity^*b*^
*Escherichia coli* ATCC 15597				+
*Escherichia coli* ATCC 25922				+
*Escherichia coli*	ESAN 2	Underground water	AK^S^, AMP^R^, AMC AUG^R^, CAZ^R^, CIP^I^, CN^S^, CTX^R^, ETP^S^, FEP^R^, FOX^R^, IMP^S^, MEM^S^, TOB^S^, TZP^S^	+
*Escherichia coli*	ESAN 4	Underground water	AK^S^, AMP^R^, AMC AUG^R^, CAZ^R^, CIP^S^, CN^S^, CTX^R^, ETP^S^, FEP^R^, FOX^R^, IMP^S^, MEM^S^, TOB^S^, TZP^S^	+
*Escherichia coli*	ESAN 11	Underground water	AK^S^, AMP^S^, AMC AUG^S^, CAZ^S^, CIP^S^, CN^S^, CTX^S^, ETP^S^, FEP^S^, FOX^S^, IMP^S^, MEM^S^, TOB^S^, TZP^S^	+
*Escherichia coli*	ESAN 31	Underground water	AK^S^, AMP^I^, AMC AUG^S^, CAZ^S^, CIP^I^, CN^S^, CTX^I^, ETP^S^, FEP^S^, FOX^S^, IMP^S^, KZ^R^, MEM^S^, TOB^S^, TZP^S^	−
*Escherichia coli*	ECAL 45	Food	AK^S^, AMP^I^, AMC AUG^S^, CAZ^S^, CIP^S^, CN^S^, CTX^R^, ETP^S^, FEP^S^, FOX^S^, IMP^S^, MEM^S^, TOB^S^, TZP^S^	+
*Escherichia coli*	ECAL 86	Food	AK^S^, AMP^S^, AMC AUG^S^, CAZ^S^, CIP^S^, CN^S^, CTX^S^, ETP^S^, FEP^S^, FOX^S^, IMP^S^, MEM^S^, TOB^S^, TZP^S^	+
*Escherichia coli*	ECAL 130	Food	AK^S^, AMP^S^, AMC AUG^S^, CAZ^S^, CIP^S^, CN^S^, CTX^S^, ETP^S^, FEP^S^, FOX^S^, IMP^S^, MEM^S^, TOB^S^, TZP^S^	+
*Escherichia coli*	ECAL 141	Food	AK^S^, AMP^R^, AMC AUG^S^, CAZ^R^, CIP^R^, CN^S^, CTX^R^, ETP^S^, FEP^R^, FOX^S^, IMP^S^, MEM^S^, TOB^S^, TZP^S^	+
*Escherichia coli*	ETRA 1	Sewage	AK^S^, AMP^S^, AMC AUG^S^, CAZ^R^, CIP^S^, CN^S^, CTX^S^, ETP^S^, FEP^S^, FOX^S^, IMP^S^, MEM^S^, TOB^S^, TZP^S^	−
*Escherichia coli*	ETRA 5	Sewage	AK^S^, AMP^S^, AMC AUG^S^, CAZ^S^, CIP^I^, CN^S^, CTX^S^, ETP^S^, FEP^I^, FOX^S^, IMP^S^, MEM^S^, TOB^S^, TZP^S^	−
*Escherichia coli*	ETRA 6	Sewage	AK^S^, AMP^R^, AMC AUG^S^, CAZ^S^, CIP^R^, CN^S^, CTX^R^, ETP^S^, FEP^I^, FOX^S^, IMP^S^, MEM^S^, TOB^S^, TZP^S^	−
*Escherichia coli*	ETRA 7	Sewage	AK^S^, AMP^S^, AMC AUG^S^, CAZ^S^, CIP^S^, CN^S^, CTX^S^, ETP^S^, FEP^S^, FOX^S^, IMP^S^, MEM^S^, TOB^S^, TZP^S^	+
*Escherichia coli*	EMS 4	Drinking water	AK^S^, AMP^I^, AMC AUG^S^, CAZ^S^, CIP^S^, CN^S^, CTX^I^, ETP^I^, FEP^S^, FOX^S^, IMP^I^, MEM^S^, TOB^S^, TZP^S^	−
*Escherichia coli*	EMS 5	Drinking water	AK^S^, AMP^S^, AMC AUG^S^, CAZ^S^, CIP^I^, CN^S^, CTX^S^, ETP^I^, FEP^S^, FOX^S^, IMP^I^, MEM^S^, TOB^S^, TZP^S^	−
*Escherichia coli*	EMS 6	Drinking water	AK^S^, AMP^S^, AMC AUG^S^, CAZ^S^, CIP^S^, CN^S^, CTX^I^, ETP^S^, FEP^I^, FOX^S^, IMP^S^, MEM^S^, TOB^S^, TZP^S^	−
*Escherichia coli*	EMS 7	Drinking water	AK^S^, AMP^I^, AMC AUG^S^, CAZ^S^, CIP^S^, CN^S^, CTX^S^, ETP^S^, FEP^S^, FOX^S^, IMP^S^, MEM^S^, TOB^S^, TZP^S^	−
*Escherichia coli*	EMS 8	Drinking water	AK^S^, AMP^I^, AMC AUG^S^, CAZ^S^, CIP^S^, CN^S^, CTX^S^, ETP^S^, FEP^I^, FOX^S^, IMP^I^, MEM^S^, TOB^S^, TZP^S^	−
*Escherichia coli*	EMS 11	Drinking water	AK^S^, AMP^I^, AMC AUG^S^, CAZ^S^, CIP^S^, CN^S^, CTX^I^, ETP^S^, FEP^I^, FOX^S^, IMP^S^, MEM^S^, TOB^S^, TZP^S^	+
*Escherichia coli*	ECO 69	Estuary	AK^S^, AMP^S^, AMC AUG^S^, CAZ^S^, CIP^S^, CN^S^, CTX^S^, ETP^S^, FEP^S^, FOX^S^, IMP^S^, MEM^S^, TOB^S^, TZP^S^	−
*Escherichia coli*	ECO 85	Estuary	AK^S^, AMP^R^, AMC AUG^I^, CAZ^R^, CIP^R^, CN^S^, CTX^S^, ETP^S^, FEP^S^, FOX^S^, IMP^I^, MEM^S^, TOB^S^, TZP^S^	+
*Escherichia coli*	ECO 119	Estuary	AK^S^, AMP^I^, AMC AUG^S^, CAZ^S^, CIP^S^, CN^S^, CTX^S^, ETP^S^, FEP^I^, FOX^S^, IMP^S^, KZ^R^, MEM^S^, TOB^S^, TZP^I^	−
*Escherichia coli*	ECO 122	Estuary	AK^S^, AMP^I^, AMC AUG^I^, CAZ^S^, CIP^S^, CN^S^, CTX^I^, ETP^S^, FEP^S^, FOX^S^, IMP^I^, MEM^S^, TOB^S^, TZP^R^	+
*Escherichia coli*	ECO 141	Estuary	AK^S^, AMP^R^, AMC AUG^I^, CAZ^S^, CIP^I^, CN^S^, CTX^S^, ETP^S^, FEP^S^, FOX^S^, IMP^S^, MEM^S^, TOB^S^, TZP^I^	+
*Escherichia coli*	ECO 142	Estuary	AK^S^, AMP^R^, AMC AUG^R^, CAZ^S^, CIP^S^, CN^S^, CTX^S^, ETP^S^, FEP^S^, FOX^S^, IMP^S^, MEM^S^, TOB^S^, TZP^I^	+
*Escherichia coli*	ECO 148	Estuary	AK^S^, AMP^R^, AMC AUG^S^, CAZ^S^, CIP^R^, CN^S^, CTX^S^, ETP^S^, FEP^S^, FOX^S^, IMP^S^, MEM^S^, TOB^S^, TZP^I^	−
*Escherichia coli*	ECO 160	Estuary	AK^S^, AMP^I^, AMC AUG^S^, CAZ^S^, CIP^I^, CN^S^, CTX^S^, ETP^S^, FEP^S^, FOX^S^, IMP^S^, MEM^S^, TOB^S^, TZP^I^	−
*Escherichia coli*	ECO 163	Estuary	AK^S^, AMP^R^, AMC AUG^I^, CAZ^S^, CIP^R^, CN^S^, CTX^I^, ETP^S^, FEP^S^, FOX^S^, IMP^S^, MEM^S^, TOB^S^, TZP^R^	−
*Salmonella enterica* subsp. *enterica* Typhimurium ATCC 14028				+
*Bacillus*				−
*Pseudomonas*				−
*Vibrio*				−

^*a*^ AK, amikacin; AMP, ampicillin; AMC AUG, amoxicillin-clavulanic acid; CAZ, ceftazidime; CIP, ciprofloxacin; CN, gentamicin; CTX, cefotaxime; ETP, ertapenem; FEP, cefepime; FOX, cefoxitin; IMP, imipenem; KZ, cefazolin; MEM, meropenem; TOB, tobramycin; TZP, piperacillin-tazobactam.

^*b*^ +, lytic; −, not lytic.

BME3 infected approximately 48% (13/27) of environmental *E. coli* strains. Among these, the infection rate was higher for antibiotic-resistant strains (67%) compared to intermediate and sensitive strains (33%). Strains were classified as antibiotic-resistant if they showed resistance to at least one antibiotic, and as intermediate/sensitive if they were either intermediate or sensitive to all tested antibiotics. Sensitive and intermediate strains were grouped as susceptible, according to the European Committee on Antimicrobial Susceptibility Testing guidelines (19). These results suggest that modifications in the bacterial surface may influence the expression of phage receptors, thereby preventing phage adsorption. Such mechanisms may incur a fitness cost on the bacteria and impact their antibiotic resistance, a phenomenon observed with other bacteriophages, such as ΦFG02, ΦC001, Phab24, and Golestan (20–22).

### Physicochemical characterization of the bacteriophage BME3

**Fig 2:**
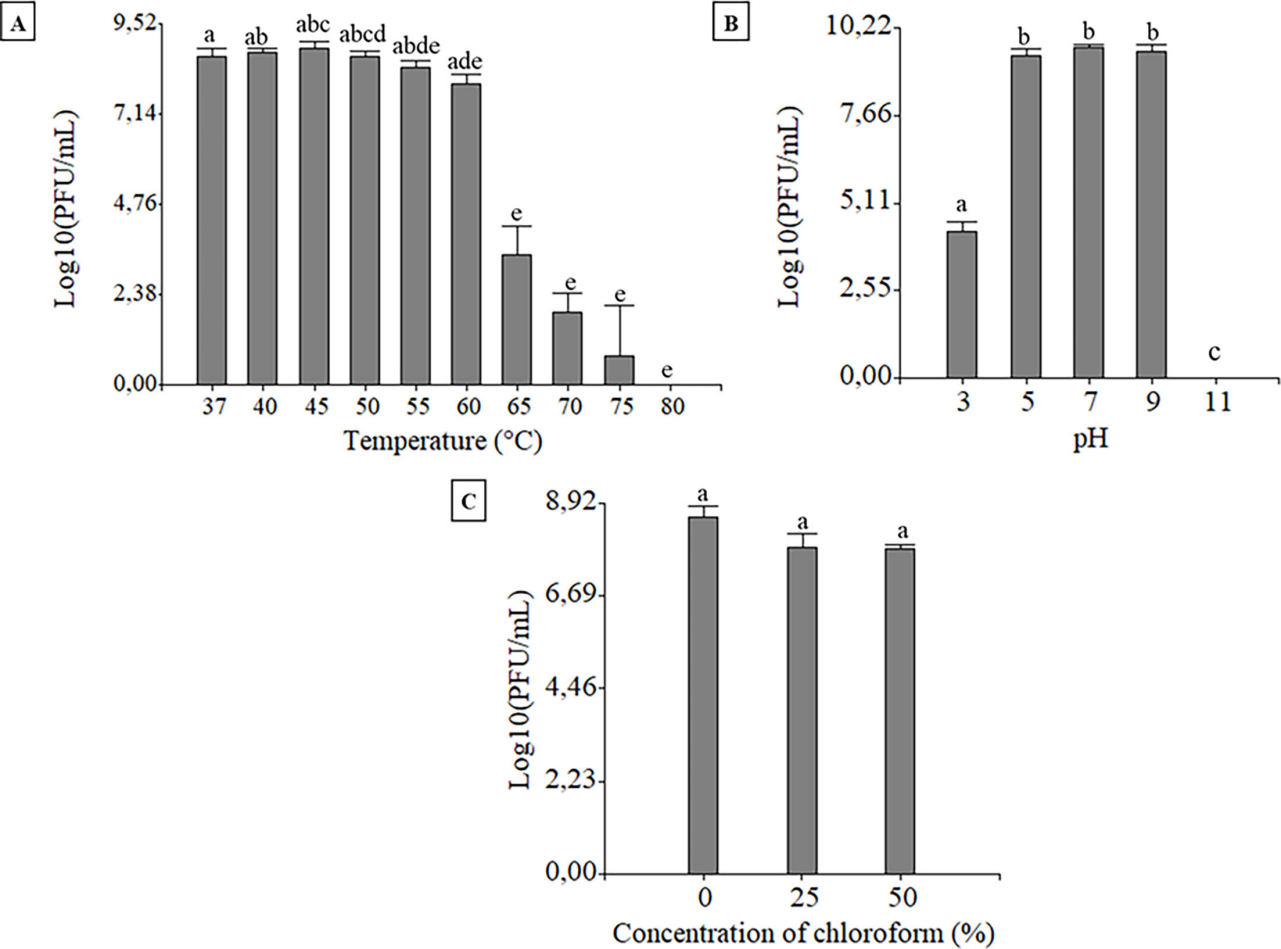
Physicochemical stability of BME3 expressed as PFU/mL. Independent experiments were done to determine the stability to temperature (**A**), pH (**B**), and chloroform (**C**). Results are presented as mean values ± standard deviations of three independent assays. Shared letters indicate that there is no statistical difference between treatments (*P* > 0.05), while different letters indicate a statistically significant difference (*P* < 0.05).

**Fig 3:**
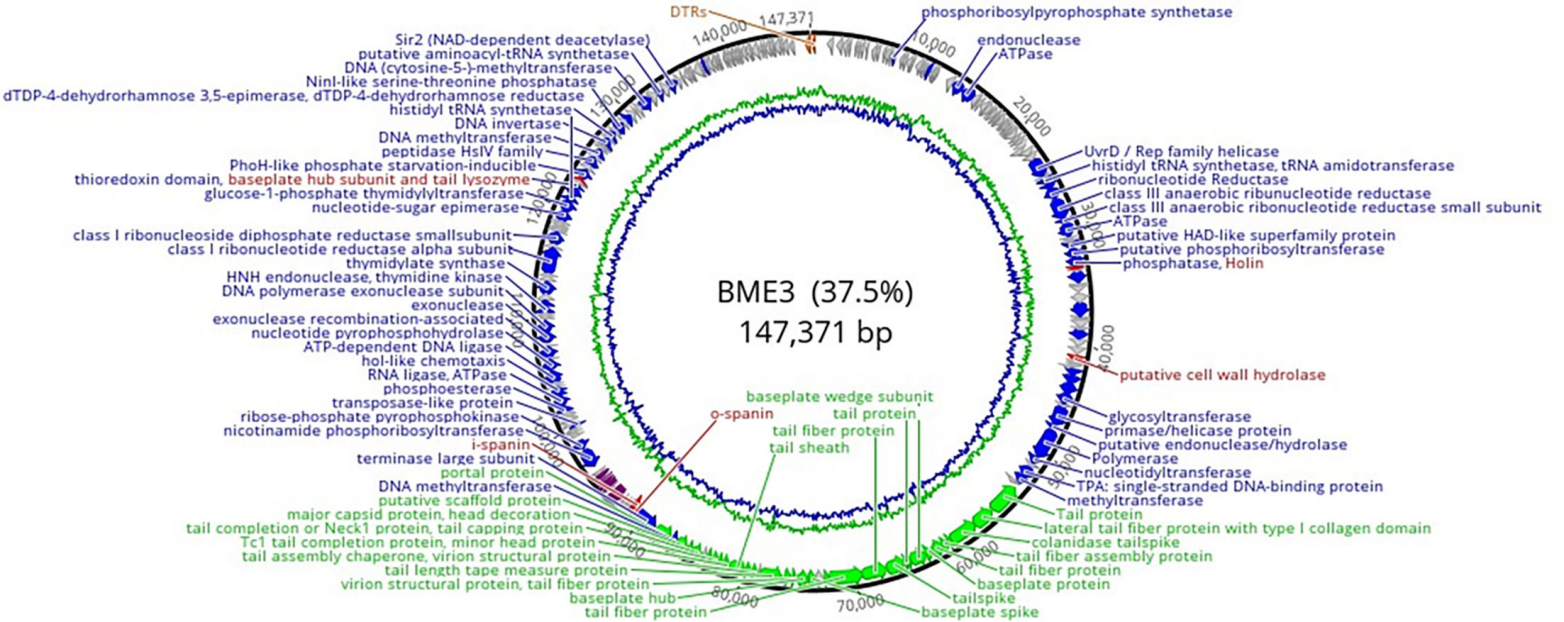
Genome map of phage BME3. The assignment of functions for each coding sequence (CDS) is as follows: structural proteins (green), replication, regulatory, nucleotide metabolism, and packaging proteins (blue), tRNA (purple), lysis (red), and hypothetical proteins (gray). Additionally, direct terminal repeats (DTRs) are included (orange).

BME3 showed stability up to a maximum temperature of 55°C for 1 h, indicating its thermostability. However, a decrease in phage titer was observed at 65°C, and temperatures above 80°C were lethal for BME3 (Fig. 2A). BME3 demonstrated stability over pH ranges between 5 and 9 for 1 h of incubation at 37°C (Fig. 2B). Similar results have been reported for T4 and T4-like phages, such as Sa45lw (23, 24). Additionally, BME3 was resistant to chloroform (Fig. 3A), like other tailed bacteriophages (25). The stability of BME3 to pH and temperature is a desirable characteristic in phages used in phage therapy, particularly for the control of *E. coli* in the environments (26–28).

### Genome annotation of the phage BME3

The complete genome of bacteriophage BME3 comprises 147,371 bp of double-stranded DNA and was deposited in NCBI with the accession number PP239276. The genome has a G+C content of 37.5% and 16 tRNAs. BME3 presented direct terminal repeats with a size of 342 bp. A total of 256 coding sequences (CDS) were identified in the genome, of which 133 CDSs were on the positive strand and 123 were on the negative strand. The function of 112 CDSs was determined by homology, while the remaining 144 CDSs were considered hypothetical proteins or proteins with unknown functions. Within the group of functional genes are structure, lysis, regulation, replication, nucleotide metabolism, and packaging proteins (Fig. 3; Table S1).

### tRNAs

BME3 contains 16 tRNA genes, corresponding to Ile^GAT^, Met^CAT^, Met^CAT^, Leu^TAG^, Phe^GAA^, Pro^TGG^, Gln^TTG^, Gly^TCC^, Thr^TGT^, Asn^GTT^, Tyr^GTA^, Lys^TTT^, Ser^GCT^, Ser^TGA^, Leu^TAA^, and Met^CAT^. These findings are consistent with those reported by Nicolas et al. (12) for the bacteriophages ESCO8, ESCO9, and ESCO32 of the *Justusliebvirus* genus, where 15 out of the 16 tRNA genes are conserved, while BME3 and ESCO8 possess an additional methionyl-tRNA copy. BME3 harbors three copies of methionyl-tRNA and two of seryl-tRNA. Serine is among the most abundant amino acids in BME3 structural proteins, whereas methionine is less frequent. However, the presence of multiple methionyl-tRNA copies is a common trait in myoviruses, hypothesized to enhance the rapid translation of phage mRNAs (29).

### DNA packaging genes

The packaging of genomic DNA involves a packaging complex comprising several proteins: terminase, which has an N-terminal ATPase domain and a C-terminal nuclease domain; and portal, which is located within the procapsid apex. The nuclease activity of the terminase cleaves the replicated DNA at the beginning and the end of the process, while the ATPase domain facilitates condensation of the packaged DNA. The interaction between the terminase and portal protein allows the storage of DNA within the capsid (30). Furthermore, the BME3 genome is predicted to contain the HNH endonuclease, which enhances terminase activity during DNA cleavage (31).

### Methylation proteins

BME3 harbors four CDSs that potentially encode protein methyltransferases, which serve to protect phage genomic material against cleavage by host restriction endonucleases (32). BLASTx analysis predicted that the phage encodes three classes of orphan DNA methyltransferase type II. These enzymes catalyze the transfer of the methyl group of S-adenosyl methionine, resulting in the generation of products such as nitrogen-4-methylcytosine, nitrogen-6-methyladenine, and carbon-5-methylcytosine (33). Furthermore, the presence of these proteins may contribute to BME3’s wide host range, ensuring its infectivity and the survival of its progeny, as observed in *Salmonella* and *Yersinia* phages (32, 34, 35).

### Nucleotide metabolism proteins

The genome of bacteriophage BME3 reveals the presence of class I (NrdA and NrdB) and class III (NrdD and NrdG) ribonucleotide reductases. These enzymes play a crucial role in deoxyribonucleotide synthesis, operating under aerobic and anaerobic conditions, respectively (36). Notably, similar findings have been reported in phage rV5 (29). Other enzymes involved in DNA synthesis are thymidylate synthase and thymidine kinase (37).

### Replication and repair proteins

Two CDSs coding helicase/primase and UvrD/Rep family helicase were predicted within the genome of bacteriophage BME3. These enzymes are involved in diverse processes such as DNA replication, repair, and recombination. The helicase/primase in hexameric form, together with the polymerase and the single-stranded DNA-binding protein (ss), integrates into the replisome or replication complex, facilitating the generation of multiple copies of the genomic material (38). Each of these enzymes requires thioredoxin as a cofactor, which is derived from the host cell. Meanwhile, the UvrD/Rep family helicase, in non-hexameric form, exhibits a 3′→ 5′ polarity to unwind DNA, and its main function is to repair genomic material (39).

### Lysis proteins

The genome of BM3 and phage AchV4 present a genetic organization in which the holin gene does not precede the endolysin gene, as usually reported in most tailed bacteriophages (40). Using DeepTMHMM 1.0.42, proteins near endolysins were determined to lack transmembrane domains. However, two small proteins (61 and 81 residues) located further away were identified that exhibit features of topology II holins (29, 35).

BM3, like PVP-SE1, encodes two endolysins: a lysozyme in the structural module and a hydrolase in the regulatory and replication module, the latter homologous to SleB, an acetylmuramidase enzyme (35, 41). BME3 endolysins have great potential in controlling antibiotic-resistant bacteria (42). Likewise, the spanin complex was identified to be encoded by two separate genes, one for the outer membrane lipoprotein (o-spanin) and one for the inner membrane protein (i-spanin) (43). Phage endolysins have great potential to control bacterial pathogens and even combat biofilm formation. These enzymes are not toxic to humans (42).

### Morphogenesis proteins

The structural proteins have three main segments: the capsid, tail, and baseplate proteins. The capsid includes the major capsid protein, head decoration protein, and scaffold protein. The proteins that integrate the tail structure are contractile sheath, central tube, and tape measure. Notably, the length of the tape measure protein correlates with the length of the tail; phi92 and BME3 exhibit the same number of residues (659 aa). Regarding the baseplate structure, it is composed of tail fibers and tail spikes (44). These proteins have an identity percentage higher than 98% (96% coverage) with bacteriophages such as phi92, muut, PHBO5, alia, and PDO6, except the tail spike protein. The sequence differences are scattered and not concentrated in a specific region.

### Phylogenetic analysis

**Fig 4:**
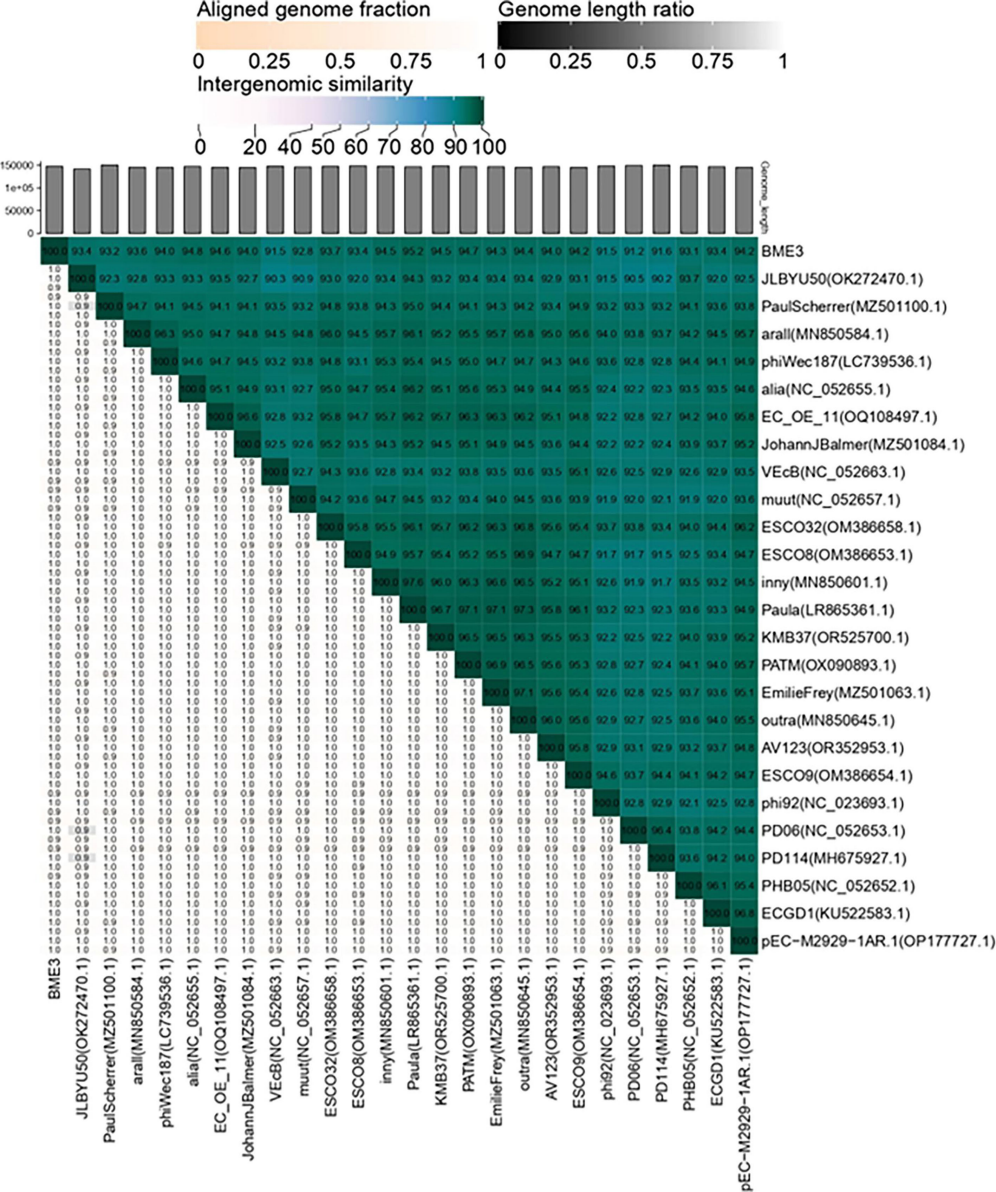
VIRIDIC heat map of BME3 with the 27 closest homologs identified by BLAST. The right half shows the intergenomic similarity between phages, where the color intensity indicates the level of similarity. The lower left half shows the percent coverage of the first phage, along with the percent alignment and percent coverage of the second phage.

The BLAST search performed at NCBI revealed homology between the phage BME3 and the genomic sequences of phi92-like phages such as KMB37, Paula, inny, alia, EmilieFrey, PHB05, arall, and muut, with identities in the 98% range (sequence coverage between 93% and 97%). The Virus Intergenomic Distance Calculator (VIRIDIC) was used to calculate the intergenomic similarity of phage BME3 and other species of the genus *Justusliebigvirus*. Overall, BME3 shows less than 95% identity with other species, except for Paula, which showed 95.2% identity (Fig. 4). Paula, an *E. coli* phage isolated from water sources around Durham (UK) in 2016, has been subjected to antiphage defense mechanisms (45, 46); however, it was not fully characterized.

**Fig 5:**
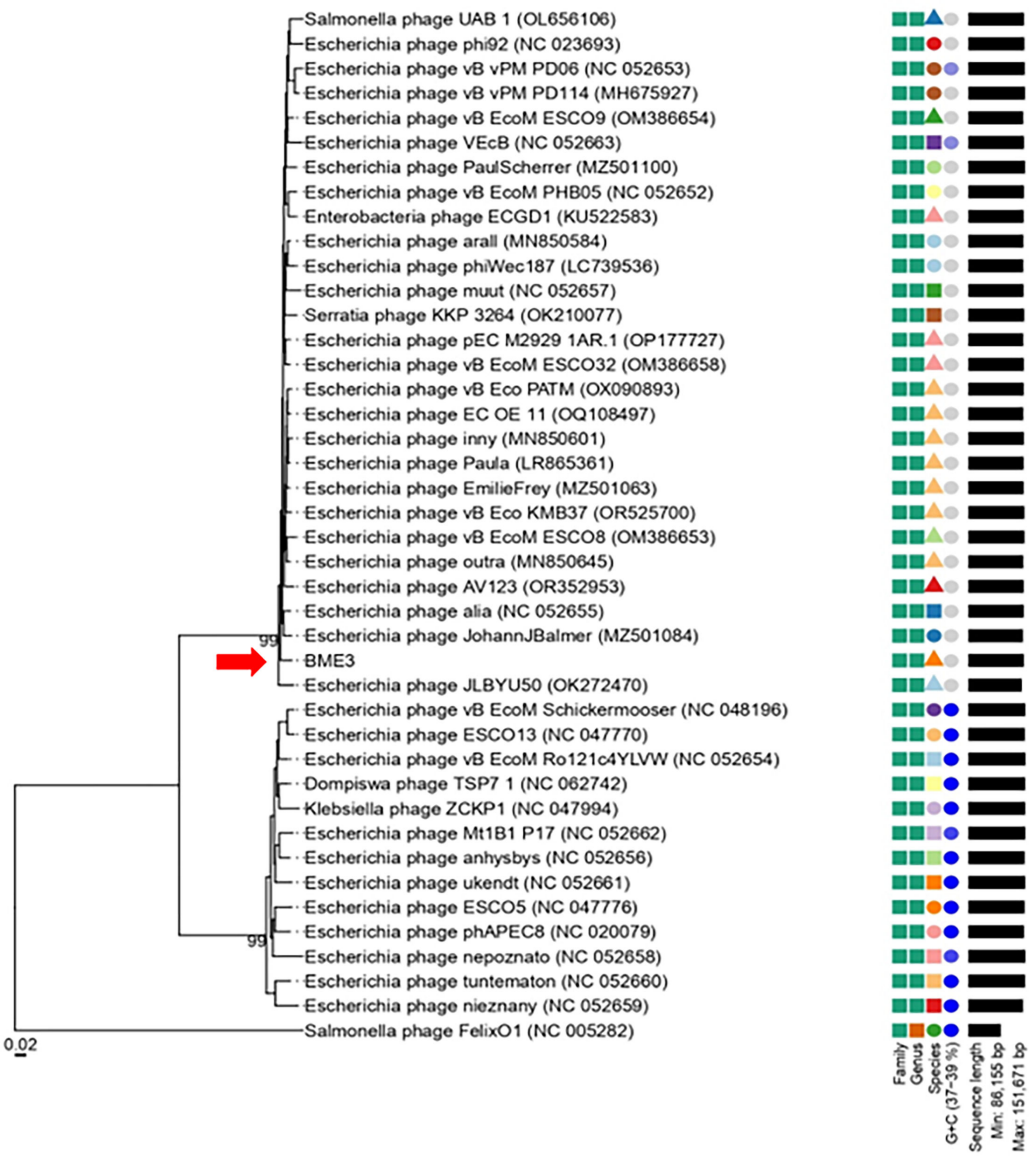
Whole genome-based phylogenetic tree of BME3 and close relatives generated by the VICTOR website. Phylogenomic Genome-BLAST Distance Phylogeny (GBDP) tree inferred using the formula D0 (nucleotide) and yielding an average support of 6%. The numbers above branches are GBDP pseudo-bootstrap support values from 100 replications. The branch lengths of the resulting VICTOR trees are scaled in terms of the respective distance formula used. The arrow points to BME3.

Subsequently, a phylogenetic tree at the nucleotide level was constructed using the VICTOR platform, confirming that BME3 belongs to the genus *Justusliebigvirus*. Additionally, phages such as Nepoznato, ESCO5, ZCKP1, and Anhysbys, which were classified within the genus *Phapecoctavirus*, were also found to be related to BME3. This supports the placement of both genera, *Justusliebigvirus* and *Phapecoctavirus*, within the subfamily *Stephanstirmvirinae* (47) (Fig. 5).

**Fig 6:**
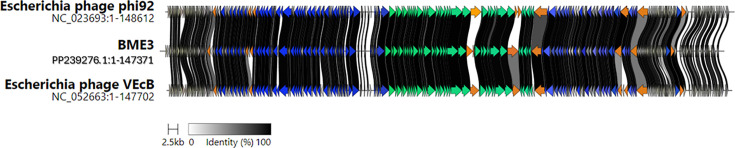
Genome comparison of bacteriophage phi92, BME3, and VEcB with Clinker. The arrows represent the coding sequences, the green color represents structural proteins, and the blue color represents replication, regulatory, and packaging proteins. The arrows are linked by shaded regions that indicate the percentage of amino acid identity. The orange dates represent proteins with percent identity other than 100% such as NAD-dependent histone deacetylase, peptidases family of HslV, PhoH-like starvation-inducible phosphate, tail spike, tail fiber, class III anaerobic ribonucleotide reductase, UvrD/Rep family helicase, and endonuclease (orange color from left to right). Among these proteins, the tail spike and tail fiber showed no conservation relative to their homologs.

BME3, along with other members of the *Justusliebigvirus* genus, has demonstrated the capacity to lyse enterobacteria, including *Escherichia coli* and *Salmonella enterica* subsp. *enterica* serovar Typhimurium (11, 12). This lytic activity could extend to encapsulated and unencapsulated strains and can be attributed to their multivariate adsorption apparatus, which compares to a nanometer Swiss army knife due to the arrangement of receptor-binding proteins (RBPs), allowing a larger contact surface and access to different bacterial receptors (11, 48). In the genome comparison, it is observed that the least conserved genes are those encoding for structural proteins, particularly receptor-binding proteins, such as tail spike protein and tail fiber (Fig. 6). The tail spike consists of two CDSs with lengths of 102 and 552 amino acids, respectively. The first segment shows similarity to members of the *Justusliebigvirus* genus (Paul Scherrer, with 79% identity and 98% coverage) and *Phapecoctavirus* (ZCKP1, with 100% identity and 98% coverage). Meanwhile, the second segment shows homology with receptor-binding proteins, GenBank accession no. MDB8464820.1, with 91% coverage and 82.7% identity, and GenBank accession no. WP_252514376.1, with 91% coverage and 82.68% identity. Furthermore, the CDS for the tail fiber is 939 amino acids long. The phage BME3 shares similarities with other phages of the *Justusliebigvirus* genus, such as PHB05, phiWec187, alia, JLBYU50, Paula, inny, and Paul Scherrer, except for phage phi92. The BLASTx results indicate a coverage and identity percentage higher than 99% and 86%, respectively.

RBPs have a homotrimeric structure and generally possess two domains: N-terminal and C-terminal. The N-terminal domain facilitates the binding of RBP to the phage baseplate and can be reused between family members. In contrast, the C-terminal domain may harbor a catalytic center with depolymerase activity and include chaperones responsible for proper protein folding and facilitating multimerization (48–51).

The tail spikes, due to their depolymerase activity, can degrade the bacterial capsule or lipopolysaccharide (LPS), which allows the phage to access the bacterial membrane and initiate infection (44, 51, 52). During infection, the lateral fibers of the phage tail bind to host surface glycans or enterobacterial common antigens. Next, the short tail fibers recognize glucose from the outer core of the LPS, causing injection of the phage genome into the bacterial cell (53).

BME3, like phi92, is a polyvalent phage, possessing a branched structure of RBPs (11). The RBPs with the highest conservation among members of the genera *Justusliebigvirus*, *Phapecoctavirus*, and *Seunavirus* are the colanidase tail spike and tail fiber (phi92 gene 141). In particular, the colanidase tail spike gene of BME3 shares 98% similarity with *Justusliebigvirus* (100% coverage), 74% with *Phapecoctavirus* (92% coverage), and 37% with *Seunavirus* (73% coverage). The tail fiber of BME3 shows 98% similarity with *Justusliebigvirus* (100% coverage), 83% with *Phapecoctaviurs* (100%), and 77% with *Seunavirus* (97% coverage). This protein conservation likely enables BME3 and its homologs to recognize *Salmonella-*specific receptors. This is supported by the fact that PVP-SE1, a member of the *Seunavirus*, exhibits a broad lytic spectrum against *Salmonella* strains (35, 54).

In contrast, the other tail spike lacks homology with the enzyme endosialidase, responsible for degrading the capsule composed of sialic acid with α−2,8 and α−2,9 bonds (44). Similarly, the last enzyme shows low conservation among members of the *Justusliebigvirus* genus (4).

BME3 encodes a depolymerase, as indicated by BLASTx results, suggesting a possible correspondence with an endorhamnosidase (endoglycosidase) based on its similarity to the tail spike protein of *Salmonella* phage P22 (55). Active site interchangeability between an endorhamnosidase and an endosialidase is feasible and can occur through horizontal gene transfer mechanisms (52, 56–58).

### PCR detection of BME3

**Fig 7:**
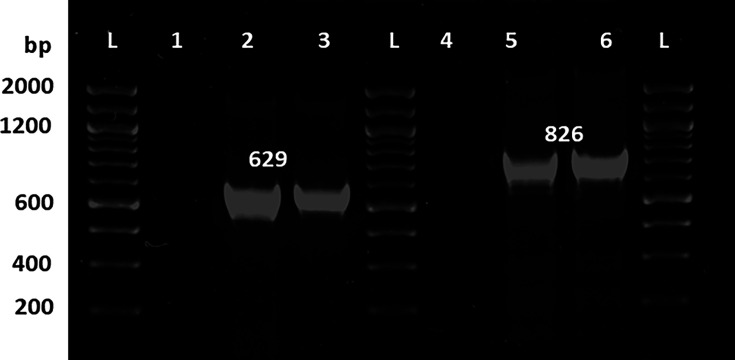
BME3 detection by polymerase chain reaction (PCR). Amplification of fragments corresponding to the major capsid protein (629 bp) and polymerase (826 bp) genes is shown. L: DNA marker (Thermo Fisher Scientific 100-2000 bp). Lanes 1 and 4: negative control; lanes 2 and 5: DNA extracted by the method of Born et al. (59); lanes 3 and 6: DNA extracted by a modified method of Jofre and Muniesa (60).

The two primer sets designed, one for amplifying the capsid protein gene and the other for the DNA polymerase gene, respectively, allowed the detection of phage BME3 out of the original sample regardless of the DNA extraction method used (Fig. 7). The primers were designed to detect BME3 and other members of the *Justusliebigvirus*, but not other phages. This was partly confirmed as no PCR amplification occurred when the primers were used on *Dhillonvirus* and *Felixounavirus* phages available in our collection (not shown).

### Conclusions

A lytic bacteriophage belonging to the genus *Justusliebigvirus* was isolated from tropical water samples collected from Estero Salado, located in the city of Guayaquil, Ecuador. This phage exhibits the ability to infect various strains of *E. coli* with different antibiotic susceptibility phenotypes, as well as *Salmonella*. BME3 has *Myoviridae* morphology, is thermostable, resistant to pH variations, and is not sensitive to chloroform. Genomic comparisons show that BME3 is related more closely to Paula phage, although limited genomic information is available for the latter. Furthermore, its genome does not harbor genes associated with antibiotic resistance, toxins, or lysogeny genes, making BME3 a potential candidate for phage therapy to mitigate the environmental spread of bacterial antibiotic resistance. To the best of our knowledge, this study is the first of its kind conducted on tropical estuarine samples collected from Ecuador.

## MATERIALS AND METHODS

### Bacterial strains and growth conditions

A total of 29 strains of *E. coli* and one of each of the following genera, *Pseudomonas* sp., *Vibrio* sp., *Bacillus* sp., and *Salmonella*, were used in this work (Table 1). *E. coli* strains were obtained from the bacterial collection in the Environmental Microbiology Laboratory, at ESPOL, isolated from environmental water and food samples. Antibiotic susceptibility phenotypes were considered in the selection. The other genera are microorganisms commonly found in water or another environment. For routine growth, bacterial strains were cultured at 37°C overnight in tryptone soy broth (TSB) or tryptone soy agar (TSA). The *E. coli* ATCC 15597 strain was used for the isolation and propagation of bacteriophages.

The antimicrobial susceptibility testing was performed using the disk diffusion method proposed by the Clinical and Laboratory Standards Institute (CLSI). A total of 14 antibiotics belonging to different groups were used. After incubation at 35°C for 16–24 h, clearance zones were measured. The interpretative criteria to determine susceptible, intermediate, and resistant, proposed by the CLSI, were applied. *E. coli* ATCC25922 was used as a control strain (61).

### Isolation, purification, and enrichment of bacteriophage

The water samples were collected from Estero Salado, a tropical estuary located in the city of Guayaquil, Ecuador (–2.4198713, –80.0299257). The sampling was non-probabilistic by convenience, consisting of 15 sampling points covering different branches of the estuary. These sampling points were selected based on criteria such as safety, accessibility, and the influence of anthropogenic activity. Bacteriophages were isolated using the single-layer agar method described by the United States Environment Protection Agency (62). Briefly, 500 µL of MgCl_2_ (4 M) and 1,000 µL of tetrazolium chloride (1%) were added to 100 mL of the water sample, followed by incubation for 10 min at 37°C. Then, 10 mL of logarithmic phase-growing *E. coli* ATCC 15597 (0.5 McFarland standard) was added to the mixture and incubated for 5 min at 37°C. Then, 100 mL of TSA (2×) previously tempered at 45°C was added to the mixture, homogenized, and dispensed into Petri dishes for subsequent incubation at 37°C for 24 h.

The recovery and purification of the bacteriophages were performed according to the methodology described by Lukman et al. (63). A single phage plaque was recovered with a sterile micropipette tip and resuspended in 1 mL of sodium magnesium buffer (1 L SM buffer: 50 mL 1 M Tris-Cl, pH 8; 2 g MgSO_4_.7H_2_O; 5.8 g NaCl). The suspension was passed through a filter of polyvinylidene fluoride membrane with a pore size of 0.22 µm. The double-layer agar method was performed at least three times until uniform lysis plates were obtained, then the phage titer was determined.

The double-layer agar method was performed following the methodology described by Kropinski et al. (64) with some modifications. The initial phage suspension was subjected to serial dilutions ranging from 10^−4^ to 10^−7^, each with a volume of 1,000 µL. Then, 200 µL of the logarithmic phase-grown bacteria (0.5 McFarland standard) and 10 µL of MgCl_2_ (4M) were added to the dilutions. The mixture was homogenized in a vortex and then incubated at 37°C for 5 min. The sample was then combined with 4 mL of 0.7% TSA prewarmed to 45°C and dispensed onto the surface of 1.5% TSA plates. The Petri dishes were incubated at 37°C for 12 h. The results were reported as PFU per milliliter of sample.

The phage titer was increased with the technique described by Poxleitner et al. (65) with some modifications. Initially, a lysis plaque was recovered using a sterile micropipette tip and resuspended in 5 mL of TSB, then 300 µL of strain ATCC 15597 (0.5 McFarland) was added to the suspension. After an incubation period of 4 h at 37°C, the mixture was centrifuged at 8,000 × *g* for 10 min and the supernatant was recovered. Next, the supernatant was filtered through a 0.22 µm membrane. Finally, the phage titer was determined using the double-layer agar method.

### Bacteriophage host range

To determine the host range of BME3 bacteriophage, the spot test was performed according to the methodology described by Kutter (66). Bacterial strains (0.5 McFarland) from the environment and food sources (Table 1) were inoculated in Petri dishes containing TSA, followed by the addition of 7 µL of each phage (1 × 10^9^ PFU/mL). ATCC 5597 was used as a control strain. Results were recorded as the presence or absence of phage lytic activity against each bacterial strain. All assays were performed in triplicate.

### Thermal and pH stability

To determine the stability of the phage under different temperature and pH conditions, the methodology described by Hu et al. (67) with some modifications was used. The thermal stability was evaluated with 1 mL of phage lysate (1 × 10^8^ PFU/mL) subjected to the following temperatures: 37°C, 40°C, 45°C, 45°C, 50°C, 55°C, 60°C, 65°C, 75°C, and 80°C for 1 h. In the pH range, 100 µL of phage lysate (1 × 10^9^ PFU/mL) was added to 900 µL of SM buffer (sodium citrate, sodium acetate, SM, and sodium carbonate) adjusted with NaOH or HCl at pH 3, 5, 7, 9, and 11. The samples were incubated at 37°C for 1 h. To determine the titer of each treatment, the double-layer agar method was employed using *E. coli* ATCC 15597 as the host strain. All assays were performed in triplicate.

### Chloroform sensitivity

To determine the chloroform sensitivity of BME3, we used the method described by Chénard et al. (68). Five hundred microliters of the purified phage (1 × 10^9^ CFU/mL) were supplemented with 500 µL of pure chloroform and shaken for 1 h. The mixture was centrifuged at 4,100 × *g* for 5 min and the supernatant was immediately recovered and incubated at 17°C for 2 h to remove residual chloroform. Phage titer was determined by the double-layer agar method, and *E. coli* ATCC 15597 was used as host. All assays were performed in triplicate.

### Examination of phage by TEM

The staining was performed with the method described by Wang et al. (69) with modifications. A volume of 5 µL of the purified bacteriophage (1 × 10^9^ PFU/mL) was deposited on a Formvar carbon supported copper grid (300 mesh, approx. grid hole size 63 µm) (PELCO catalog number 01,753-F). Grids were stained with 5 µL of 2% phosphotungstic acid for 1 s; the excess solution was removed with filter paper. The morphology of the phages was examined with TEM (FEI brand Tecnai G2 Spirit Twin model, equipped with Eagle 4K camera), operating at 80 kV. Seventeen viral particles were measured using an image analysis software, Fiji version 1.54f, to determine the tail length and capsid diameter.

### DNA extraction and genome sequencing

The DNA extraction method used was the one described by Jofre and Muniesa (60) with some modifications. Five hundred microliters of phage suspension (1 × 10^9^ PFU/mL) was treated with 1.5 µL DNAse I (6 U/µL) (Applied Biological Materials Inc., Canada) and 0.5 µL RNAse (20 ug/mL) (Invitrogen, USA) at 37°C for 1 h. The DNase I was inactivated by incubating the mixture at 80°C for 15 min. After adding 12 µL proteinase K (20 mg/mL) (Invitrogen, USA) and 1,000 µL proteinase K buffer (1M Tris HCl, pH 8, 2 mL 0.5 M EDTA, pH, 10 mL 10% SDS, 100 mL H_2_O), the mixture was incubated at 55°C for 1 h. The DNA was extracted and purified using DNA-binding columns (OMEGA BIO-TEK, USA).

The DNA was visualized in agarose (0.8%) gel electrophoresis and quantified using an ultraviolet-visible spectrophotometer (NanoDrop). The phage genome was sequenced using the Illumina NovaSeq6000 system as 150 nt paired-end reads.

### Genome assembly and bioinformatics analysis

Sequence analyses including normalization, *de novo* assembly, and annotations were performed using BBNorm, SPAdes (70), and Glimmer3 (71) in Geneious Prime version 2023.2.1. DNA packaging was determined using PhageTerm (72). Gene annotation was performed using BLAST and Uniprot tools at NCBI and RCSB Protein Data Bank (73). Transfer RNAs (tRNAs) were searched using tRNA scan-SE (74) and ARAGORN (75). CARD (76) and ResFinder 4.1 1 (77) databases were used to identify antimicrobial-resistant genes, and the PhageScope database (78) for detecting potential virulence factors present in the phage genome. PhageScope and DeepTMHMM platforms were used to predict proteins with transmembrane topology (78, 79).

Phylogenetic analysis and taxonomic classification of phage were inferred by nucleotide-level phylogenetic tree using the VICTOR web server (80) to assist in the taxonomic classification of the virus and VIRIDIC (81) platforms. Furthermore, comparative coding regions (CDS) analysis was run between related phages using Clinker (82).

### Primer design and PCR detection

The major capsid protein (MCP) and DNA polymerase (Pol) genes were used for designing detection primers to ensure the purity of the phage lysates. The primers were designed from a multiple sequence alignment (MSA) built with homologs from members of the *Justusliebigvirus* genus. MSA and primer design were done in Geneious Prime, and their genus-level specificity was checked by BLASTn (Table 2).

**TABLE 2:** Detection primers^*a*^

Name	Sequence (5´→3´)	Product size (bp)
MCP-fw	CAGACTGTAGCTGTACAGTGCG	629
MCP-rev	TGATAACCTGCGTACCGAAGCG	
Pol-fw	GGTATGGAGCCACCATCGCACT	826
Pol-rev	GCCTTACGAACAAGATCATCGTC	

^*a*^ Forward (fw) and reverse (rev) primers for the amplification of a fragment of the MCP and the DNA polymerase.

In this experiment, the DNA template obtained through the modified methodology detailed by Jofre and Muniesa (60) was used. Furthermore, the method proposed by Born et al. (59) was applied, as described below.

A lytic plate was recovered with a sterile micropipette tip and resuspended in 100 µL of SM buffer. Then, the phage suspension was incubated at room temperature (approximately 17°C) for 1 h, followed by incubation at 95°C for 10 min. The sample was then quickly refrigerated at 8°C.

The PCR mixture contained 1 µL of template DNA, 5 µL of GoTaq 2× Green Master Mix (Promega, USA), 0.5 µL of each primer at 40 µM (Macrogen, South Korea), and 3 µL of molecular grade ddH_2_O (Invitrogen, USA). Thermocycling parameters were as follows: an initial denaturation step at 95°C for 4 min, 35 cycles of 95°C × 40 s, 58°C × 30 s, and 72°C × 45 s, followed by a final extension at 72°C for 7 min. The amplified products were separated by electrophoresis on 2% agarose gels and visualized under UV light.

### Statistical analysis

The physicochemical characterization tests were subjected to parametric analysis; particularly, the Shapiro-Wilk test was used to confirm the normal distribution. Data were subjected to a one-way analysis of variance followed by Tukey’s *post hoc* comparisons to check the differences between treatments. All the analyses were performed with a significance level of 0.05, using the statistical software R version 4.2.2 and Infostat version 2020.

## Data Availability

The genome sequence corresponding to the bacteriophage reported in this study is available in GenBank under accession number PP239276.1.

## SUPPLEMENTAL MATERIAL

Table S1 spectrum.01301-24-s0001.docx Bacteriophage genome annotation.

ASM does not own the copyrights to Supplemental Material that may be linked to, or accessed through, an article. The authors have granted ASM a non-exclusive, world-wide license to publish the Supplemental Material files. Please contact the corresponding author directly for reuse.
