# Supplementary material for: Physicochemical, genomic, and phenotypic characterization of Escherichia phage BME3
Source: Microbiol Spectr. 2025 May 22;13(7):e01301-24. doi: 10.1128/spectrum.01301-24 (PMC12210895; doi:10.1128/spectrum.01301-24)
Supplement: Table S1 — Bacteriophage genome annotation. [file spectrum.01301-24-s0001.docx]

**SUPPLEMENTAL MATERIAL**

**Table 1.** Bacteriophage genome annotation

| Minimum | Maximum | Direction | Lenght (aa) | Putative function | Best-matchBLASTp Result | Query cover (%) | E-values | Identify (%) | Accession | MW (kDa) |
| --- | --- | --- | --- | --- | --- | --- | --- | --- | --- | --- |
| 1483 | 1866 | Reverse | 127 | Hypothetical protein | *Escherichia* phage phi92 | 96 | 4E-90 | 100 | YP_009012581.1 | 14.778 |
| 2327 | 2752 | Reverse | 141 | Hypothetical protein | *Escherichia* phage vB_vPM_PD114 | 99 | 2.00E-87 | 99.29 | AXY81601.1 | 16.492 |
| 2806 | 3192 | Reverse | 128 | Hypothetical protein | *Escherichia* phage alia | 99 | 6.00E-78 | 98.44 | YP_009985227.1 | 14.174 |
| 3189 | 3413 | Reverse | 74 | Hypothetical protein | *Escherichia* phage EC_OE_11 | 94 | 2.00E-42 | 98.59 | WBF54253.1 | 8.507 |
| 3784 | 4053 | Reverse | 89 | Hypothetical protein | *Escherichia* phage arall | 98 | 1.00E-57 | 97.75 | QHR67553.1 | 9.905 |
| 4102 | 4272 | Reverse | 56 | Hypothetical protein | *Escherichia* phage phAPEC8 | 98 | 7.00E-33 | 100 | YP_007348377.1 | 6.433 |
| 4354 | 4638 | Reverse | 94 | Hypothetical protein | *Escherichia* phage alia | 98 | 1.00E-61 | 100 | YP_009985222.1 | 10.966 |
| 4721 | 5044 | Reverse | 107 | Hypothetical protein | *Escherichia* phage alia | 99 | 1.00E-71 | 98.13 | YP_009985221.1 | 12.492 |
| 5374 | 5676 | Reverse | 100 | Hypothetical protein | *Escherichia* phage phi92 | 99 | 1.00E-52 | 100 | YP_009012573.1 | 11.015 |
| 5724 | 6125 | Reverse | 133 | Hypothetical protein | *Escherichia* phage phi92 | 99 | 3.00E-90 | 99.25 | YP_009012572.1 | 14.216 |
| 6435 | 6572 | Reverse | 45 | Hypothetical protein | *Escherichia* phage phiWec187 | 97 | 7.00E-22 | 100 | BDU13113.1 | 5.628 |
| 6588 | 6947 | Reverse | 119 | Hypothetical protein | Enterobacteria phage ECGD1 | 99 | 2.00E-79 | 99.16 | AMM43335.1 | 14.158 |
| 7034 | 7204 | Reverse | 56 | Hypothetical protein | *Escherichia* phage phi92 | 98 | 1.00E-20 | 100 | YP_009012568.1 | 6.091 |
| 7296 | 7511 | Reverse | 71 | phosphoribosylpyrophosphate synthetase | *Salmonella* phage UAB_1 | 98 | 2.00E-41 | 100 | UIS31601.1 | 8.164 |
| 8004 | 8243 | Reverse | 79 | Hypothetical protein | *Escherichia* phage alia | 98 | 7.00E-51 | 100 | YP_009985215.1 | 9.080 |
| 8329 | 8511 | Reverse | 60 | Hypothetical protein | *Escherichia* phage vB_EcoM_PHB05 | 98 | 6.00E-35 | 100 | YP_009984590.1 | 6.718 |
| 8600 | 8965 | Reverse | 121 | Hypothetical protein | *Escherichia* phage VEcB | 99 | 1.00E-83 | 100 | YP_009987438.1 | 13.716 |
| 9061 | 9240 | Reverse | 59 | Hypothetical protein | *Escherichia* phage alia | 98 | 5.00E-31 | 98.31 | YP_009985213.1 | 6.923 |
| 9388 | 9942 | Reverse | 184 | Hypothetical protein | *Escherichia* phage PaulScherrer | 99 | 9.00E-100 | 98.91 | QXV83854.1 | 20.867 |
| 9944 | 10204 | Reverse | 86 | Hypothetical protein | *Escherichia* phage phiWec187 | 98 | 5.00E-57 | 98.84 | BDU13105.1 | 9.763 |
| 10207 | 10353 | Reverse | 48 | Hypothetical protein | *Escherichia* phage phi92 | 97 | 2.00E-26 | 100 | YP_009012561.1 | 5.537 |
| 10432 | 10794 | Reverse | 120 | Membrane protein | *Escherichia* phage JLBYU50 | 99 | 9.00E-83 | 97.5 | UGL62073.1 | 13.909 |
| 10880 | 11152 | Reverse | 90 | Hypothetical protein | *Escherichia* phage vB_EcoM_PHB05 | 98 | 2.00E-57 | 97.78 | YP_009984586.1 | 9.877 |
| 11211 | 11354 | Reverse | 47 | Hypothetical protein | *Salmonella* phage UAB_1 | 79 | 4.00E-17 | 97.37 | UIS31592.1 | 5.660 |
| 11402 | 11614 | Forward | 70 | Hypothetical protein | *Salmonella* phage UAB_1 | 98 | 5.00E-43 | 97.14 | UIS31591.1 | 7.750 |
| 12428 | 12979 | Reverse | 183 | Hypothetical protein | *Escherichia* phage VEcB | 99 | 5.00E-132 | 100 | YP_009987431.1 | 21.738 |
| 12980 | 13969 | Reverse | 329 | Endonuclease | *Escherichia* phage VEcB | 98 | 0 | 100 | YP_009987430.1 | 37.513 |
| 14044 | 15171 | Reverse | 375 | ATPase | *Escherichia* phage phi92 | 98 | 0 | 100 | YP_009012554.1 | 41.748 |
| 15183 | 15620 | Reverse | 145 | Hypothetical protein | *Escherichia* phage alia | 99 | 1.00E-89 | 99.31 | YP_009985203.1 | 16.473 |
| 15635 | 15808 | Reverse | 57 | Hypothetical protein | *Escherichia* phage phi92 | 92 | 3.00E-29 | 100 | YP_009012552.1 | 6.644 |
| 15829 | 16080 | Reverse | 83 | Hypothetical protein | *Escherichia* phage phi92 | 98 | 1.00E-50 | 98.8 | YP_009012551.1 | 9.535 |
| 16080 | 16631 | Reverse | 183 | Hypothetical protein | *Escherichia* phage phiWec187 | 99 | 7.00E-131 | 97.27 | BDU13095.1 | 21.656 |
| 16751 | 17041 | Forward | 96 | Hypothetical protein | *Escherichia* phage JohannJBalmer | 98 | 1.00E-61 | 98.96 | QXV81445.1 | 10.803 |
| 17054 | 17341 | Forward | 95 | Hypothetical protein | *Escherichia* phage phAPEC8 | 98 | 7.00E-62 | 100 | YP_007348612.1 | 10.779 |
| 17338 | 17691 | Forward | 117 | Hypothetical protein | *Escherichia* phage phi92 | 99 | 4.00E-80 | 99.15 | YP_009012547.1 | 13.887 |
| 17704 | 17859 | Forward | 51 | Hypothetical protein | *Escherichia* phage phi92 | 98 | 9.00E-29 | 98.04 | YP_009012546.1 | 5.938 |
| 17856 | 18056 | Forward | 66 | Hypothetical protein | *Escherichia* phage muut | 98 | 1.00E-39 | 100 | YP_009985774.1 | 7.602 |
| 18057 | 18203 | Forward | 48 | Hypothetical protein | *Escherichia* phage vB_EcoM_ESCO32 | 97 | 9.00E-24 | 97.92 | UPW38609.1 | 5.416 |
| 18200 | 18628 | Forward | 142 | Hypothetical protein | *Escherichia* phage vB_EcoM_PHB05 | 99 | 2.00E-101 | 99.3 | YP_009984571.1 | 16.522 |
| 18632 | 18904 | Forward | 90 | Hypothetical protein | *Escherichia* phage vB_EcoM_PHB05 | 98 | 1.00E-58 | 98.89 | YP_009984570.1 | 11.065 |
| 18934 | 19059 | Forward | 41 | Hypothetical protein | *Escherichia* phage vB_EcoM_PHB05 | 97 | 5.00E-20 | 97.56 | YP_009984569.1 | 4.464 |
| 19069 | 19335 | Forward | 88 | Hypothetical protein | *Escherichia* phage alia | 98 | 5.00E-36 | 100 | YP_009985191.1 | 10.809 |
| 19396 | 19560 | Forward | 54 | Hypothetical protein | *Escherichia* phage phi92 | 98 | 1.00E-30 | 100 | YP_009012540.1 | 6.256 |
| 19571 | 19864 | Forward | 97 | Hypothetical protein | *Escherichia* phage VEcB | 94 | 2.00E-61 | 98.92 | YP_009987413.1 | 11.699 |
| 19866 | 20321 | Forward | 151 | Hypothetical protein | Enterobacteria phage phi92 | 99 | 7.00E-108 | 99.34 | CAH7774764.1 | 17.684 |
| 20323 | 20685 | Forward | 120 | Hypothetical protein | *Escherichia* phage phi92 | 99 | 1.00E-70 | 100 | YP_009012537.1 | 13.784 |
| 20686 | 21036 | Forward | 116 | Hypothetical protein | *Escherichia* phage phi92 | 99 | 1.00E-80 | 100 | YP_009012536.1 | 13.358 |
| 21038 | 21424 | Forward | 128 | DUF4326 domain-containing protein | *Escherichia* phage vB_vPM_PD06 | 99 | 2.00E-84 | 100 | YP_009984643.1 | 15.068 |
| 21402 | 21767 | Forward | 121 | Hypothetical protein | *Escherichia* phage vB_EcoM_PHB05 | 99 | 8.00E-82 | 99.17 | YP_009984562.1 | 13.720 |
| 21789 | 22304 | Forward | 171 | Hypothetical protein | *Escherichia* phage vB_Eco_PATM | 99 | 1.00E-124 | 99.42 | CAH7774759.1 | 20.422 |
| 22313 | 22789 | Forward | 158 | Hypothetical protein | *Escherichia* phage vB_vPM_PD06 | 99 | 4.00E-111 | 99.37 | YP_009984646.1 | 18.631 |
| 22791 | 24527 | Forward | 578 | UvrD / Rep family helicase | *Escherichia* phage EmilieFrey | 99 | 0 | 99.83 | QXV78209.1 | 65.267 |
| 24538 | 24789 | Forward | 83 | Hypothetical protein | *Escherichia* phage vB_EcoM_PHB05 | 98 | 5.00E-52 | 97.59 | YP_009984558.1 | 9.363 |
| 24786 | 25358 | Forward | 190 | Histidyl tRNA synthetase | *Escherichia* phage EC_OE_11 | 99 | 2.00E-137 | 99.47 | WBF54203.1 | 21.524 |
| 25371 | 25805 | Forward | 144 | tRNA amidotransferase | *Escherichia* phage phi92 | 99 | 3.00E-99 | 100 | YP_009012528.1 | 16.175 |
| 25823 | 26773 | Forward | 316 | Hypothetical protein | *Escherichia* phage arall | 98 | 0 | 99.68 | QHR67505.1 | 36.575 |
| 26863 | 28662 | Forward | 599 | Class III anaerobic ribunucleotide reductase | *Enterobacteria* phage ECGD1 | 99 | 0.00E+00 | 100 | AMM43526.1 | 67.367 |
| 28659 | 29129 | Forward | 156 | Class III anaerobic ribonucleotide reductase small subunit | *Escherichia* phage vB_EcoM_PHB05 | 99 | 1.00E-110 | 98.72 | YP_009984553.1 | 18.013 |
| 29150 | 30310 | Forward | 386 | ATPase | *Escherichia* phage muut | 98 | 0.00E+00 | 100 | YP_009985792.1 | 43.468 |
| 30357 | 30548 | Forward | 63 | Hypothetical protein | *Escherichia* phage phi92 | 98 | 1.00E-35 | 100 | YP_009012521.1 | 7.150 |
| 30560 | 30715 | Forward | 51 | Hypothetical protein | *Escherichia* phage phi92 | 92 | 2.00E-27 | 100 | YP_009012520.1 | 6.061 |
| 30718 | 31374 | Forward | 218 | Haloacid dehydrogenase | *Escherichia* phage PaulScherrer | 99 | 3.00E-148 | 100 | QXV83807.1 | 20.315 |
| 31367 | 32224 | Forward | 285 | Putative phosphoribosyltransferase | *Escherichia* phage vB_EcoM_PHB05 | 99 | 0.00E+00 | 98.6 | YP_009984548.1 | 33.225 |
| 32217 | 32933 | Forward | 238 | phosphatase | *Escherichia* phage alia | 99 | 1.00E-172 | 99.58 | YP_009985169.1 | 26.192 |
| 32943 | 33149 | Forward | 68 | Holin | *Escherichia* phage phi92 | 98 | 1.00E-29 | 100 | YP_009012516.1 | 7.663 |
| 33164 | 33412 | Forward | 82 | Hypothetical protein | *Escherichia* phage PaulScherrer | 98 | 2.00E-51 | 100 | QXV83803.1 | 9.081 |
| 33490 | 34467 | Forward | 325 | Tellurium resistance protein TerC | *Escherichia* phage vB_vPM_PD06 | 99 | 0.00E+00 | 100 | YP_009984663.1 | 35.753 |
| 34507 | 34713 | Forward | 68 | Hypothetical protein | *Escherichia* phage phi92 | 94 | 4.00E-41 | 100 | YP_009012513.1 | 8.119 |
| 34700 | 35290 | Forward | 196 | Hypothetical protein | *Escherichia* phage VEcB | 82 | 2.00E-30 | 99.38 | YP_009987386.1 | 20.968 |
| 35326 | 36177 | Forward | 283 | Hypothetical protein | *Escherichia* phage phiWec187 | 99 | 0.00E+00 | 99.29 | BDU13057.1 | 32.676 |
| 36246 | 37349 | Forward | 367 | tellurite resistance | *Escherichia* phage vB_EcoM_PHB05 | 99 | 0.00E+00 | 100 | YP_009984540.1 | 41.260 |
| 37363 | 37569 | Forward | 68 | Hypothetical protein | *Escherichia* phage vB_EcoM_PHB05 | 98 | 4.00E-41 | 100 | YP_009984539.1 | 7.945 |
| 37569 | 37769 | Forward | 66 | Hypothetical protein | *Escherichia* phage phi92 | 93 | 3.00E-40 | 100 | YP_009012508.1 | 7.678 |
| 37769 | 38332 | Forward | 187 | Hypothetical protein | *Escherichia* phage vB_EcoM_PHB05 | 99 | 1.00E-133 | 98.4 | YP_009984537.1 | 21.188 |
| 38325 | 38594 | Forward | 89 | Hypothetical protein | *Escherichia* phage VEcB | 98 | 3.00E-46 | 96.63 | YP_009987380.1 | 10.178 |
| 38596 | 39390 | Forward | 264 | vWA domain-containing protein | *Escherichia* phage vB_vPM_PD06 | 99 | 0.00E+00 | 100 | YP_009984671.1 | 29.618 |
| 39463 | 40059 | Forward | 198 | Hypothetical protein | *Serratia* phage KKP_3263 | 78 | 5.00E-141 | 100 | UES35883.1 | 22.090 |
| 40124 | 40726 | Forward | 200 | Hypothetical protein | *Salmonella* phage UAB_1 | 99 | 1.00E-125 | 100 | UIS31540.1 | 22.980 |
| 40819 | 41328 | Forward | 169 | Putative cell wall hydrolase | *Escherichia* phage EC_OE_11 | 99 | 4.00E-121 | 98.82 | WBF54178.1 | 19.490 |
| 41339 | 41617 | Forward | 92 | Hypothetical protein | *Escherichia* phage vB_EcoM_PHB05 | 98 | 6.00E-60 | 100 | YP_009984531.1 | 10.739 |
| 41617 | 41847 | Forward | 76 | Hypothetical protein | *Escherichia* phage phi92 | 98 | 1.00E-46 | 100 | YP_009012500.1 | 8.727 |
| 41825 | 42019 | Forward | 64 | Hypothetical protein | *Escherichia* phage phi92 | 98 | 2.00E-39 | 100 | YP_009012499.1 | 7.434 |
| 42077 | 42640 | Forward | 187 | Glycosyltransferase | *Escherichia* phage phi92 | 99 | 3.00E-135 | 100 | YP_009012498.1 | 21.562 |
| 42650 | 43336 | Forward | 228 | Glycosyltransferase | *Enterobacteria* phage ECGD1 | 99 | 3.00E-167 | 99.56 | AMM43500.1 | 26.487 |
| 43323 | 44297 | Forward | 324 | Glycosyltransferase | *Escherichia* phage vB_EcoM_PHB05 | 99 | 0 | 99.69 | YP_009984526.1 | 36.498 |
| 44309 | 45253 | Forward | 314 | Glycosyltransferase | *Escherichia* phage vB_vPM_PD114 | 99 | 0.00E+00 | 100 | AXY81524.1 | 36.183 |
| 45253 | 45522 | Forward | 89 | Hypothetical protein | *Escherichia* phage phi92 | 98 | 4.00E-57 | 100 | YP_009012494.1 | 10.828 |
| 45535 | 47307 | Forward | 590 | Primase/helicase protein | *Escherichia* phage vB_EcoM_PHB05 | 99 | 0 | 99.83 | YP_009984523.1 | 66.756 |
| 47318 | 47713 | Forward | 131 | Putative endonuclease/hydrolase | *Escherichia* phage vB_vPM_PD06 | 99 | 4.00E-90 | 99.24 | YP_009984684.1 | 14.492 |
| 47804 | 50383 | Forward | 859 | Polymerase | *Escherichia* phage vB_Eco_PATM | 99 | 0 | 99.88 | CAH7774719.1 | 98.538 |
| 50521 | 51114 | Forward | 197 | Nucleotidyltransferase | *Escherichia* phage phi92 | 99 | 2.00E-145 | 100 | YP_009012490.1 | 23.419 |
| 51158 | 51556 | Forward | 132 | Hypothetical protein | *Escherichia* phage vB_EcoM_PHB05 | 99 | 4.00E-89 | 100 | YP_009984518.1 | 15.161 |
| 51573 | 52520 | Forward | 315 | TPA: single-stranded DNA-binding protein | *Escherichia* phage outra | 99 | 0.00E+00 | 100 | QHR75395.1 | 34.416 |
| 52581 | 53312 | Forward | 243 | methyltransferase | *Escherichia* phage vB_vPM_PD06 | 99 | 0.00E+00 | 100 | YP_009984690.1 | 27.926 |
| 53269 | 53700 | Forward | 143 | Hypothetical protein | *Escherichia* phage phi92 | 99 | 4.00E-99 | 100 | YP_009012486.1 | 16.034 |
| 53736 | 56555 | Reverse | 939 | Tail protein | *Escherichia* phage vB_EcoM_PHB05 | 99 | 0 | 98.83 | YP_009984514.1 | 99.791 |
| 56595 | 58556 | Reverse | 653 | Lateral tail fiber protein with type I collagen domain | *Escherichia* phage vB_EcoM_ESCO32 | 99 | 0.00E+00 | 99.08 | UPW38668.1 | 69.088 |
| 58597 | 61011 | Reverse | 804 | colanidase tailspike | *Escherichia* phage vB_vPM_PD06 | 99 | 0 | 99.63 | YP_009984694.1 | 86.764 |
| 61011 | 61352 | Reverse | 113 | Lipoprotein | *Escherichia* phage phi92 | 99 | 3.00E-73 | 100 | YP_009012481.1 | 13.072 |
| 61362 | 61913 | Reverse | 183 | Tail fiber assembly protein | *Escherichia* phage muut | 99 | 2.00E-129 | 99.45 | YP_009985833.1 | 20.958 |
| 61923 | 62906 | Reverse | 327 | Tail fiber protein | Salmonella phage UAB_1 | 99 | 0.00E+00 | 100 | UIS31517.1 | 35.608 |
| 62920 | 63549 | Reverse | 209 | Baseplate protein | *Escherichia* phage vB_EcoM_PHB05 | 99 | 3.00E-152 | 100 | YP_009984508.1 | 23.299 |
| 63552 | 65039 | Reverse | 495 | Baseplate wedge subunit | *Escherichia* phage alia | 99 | 0.00E+00 | 100 | YP_009985129.1 | 53.981 |
| 65039 | 65284 | Reverse | 81 | Membrane protein/ holin | *Escherichia* phage vB_EcoM_PHB05 | 98 | 4.00E-52 | 100 | YP_009984506.1 | 9.468 |
| 65281 | 65589 | Reverse | 102 | Tail spike protein | *Klebsiella* phage ZCKP1 | 99 | 1.00E-67 | 100 | YP_009803406.1 | 10.765 |
| 65639 | 67297 | Reverse | 552 | Tail spike protein |  |  |  |  |  | 59.159 |
| 67306 | 69465 | Reverse | 719 | Tail fiber protein | *Escherichia* phage alia | 99 | 0.00E+00 | 99.03 | YP_009985125.1 | 81.619 |
| 69474 | 72350 | Reverse | 958 | Tail fiber protein | *Escherichia* phage vB_EcoM_ESCO8 | 99 | 0 | 99.58 | UPW37476.1 | 108.443 |
| 72350 | 72823 | Reverse | 157 | Tail fiber protein | *Escherichia* phage phi92 | 99 | 9.00E-108 | 100 | YP_009012472.1 | 16.743 |
| 72816 | 73451 | Reverse | 211 | Hypothetical protein | *Escherichia* phage vB_EcoM_PHB05 | 99 | 3.00E-138 | 100 | YP_009984499.1 | 23.635 |
| 73455 | 74192 | Reverse | 245 | Baseplate spike | *Escherichia* phage vB_EcoM_PHB05 | 99 | 9.00E-177 | 100 | YP_009984498.1 | 25.732 |
| 74195 | 75205 | Reverse | 336 | Baseplate hub | *Escherichia* phage vB_EcoM_PHB05 | 99 | 0.00E+00 | 100 | YP_009984497.1 | 37.016 |
| 75222 | 75641 | Reverse | 139 | Virion structural protein | *Escherichia* phage alia | 99 | 5.00E-84 | 99.28 | YP_009985119.1 | 15.999 |
| 75653 | 76363 | Reverse | 236 | Tail fiber protein | *Escherichia* phage vB_vPM_PD06 | 99 | 3.00E-149 | 100 | YP_009984710.1 | 26.069 |
| 76479 | 78458 | Reverse | 659 | Tail length tape measure protein | *Escherichia* phage JLBYU50 | 97 | 0.00E+00 | 99.38 | UGL62160.1 | 72.876 |
| 78478 | 78651 | Reverse | 57 | Hypothetical protein | *Escherichia* phage vB_EcoM_PHB05 | 98 | 8.00E-32 | 98.25 | YP_009984493.1 | 6.757 |
| 78753 | 79235 | Reverse | 160 | Tail assembly chaperone | *Escherichia* phage phi92 | 99 | 3.00E-112 | 100 | YP_009012464.1 | 18.092 |
| 79279 | 79758 | Reverse | 159 | Virion structural protein | *Escherichia* phage phi92 | 99 | 4.00E-110 | 99.37 | YP_009012463.1 | 17.326 |
| 79817 | 81223 | Reverse | 468 | Tail sheath | *Escherichia* phage phi92 | 96 | 0.00E+00 | 100 | YP_009012462.1 | 51.103 |
| 81244 | 81900 | Reverse | 218 | Tc1 tail completion protein | *Escherichia* phage vB_EcoM_ESCO9 | 99 | 2.00E-156 | 99.08 | UPW37690.1 | 25.038 |
| 81893 | 82309 | Reverse | 138 | Minor head protein | *Escherichia* phage phi92 | 89 | 1.00E-87 | 100 | YP_009012460.1 | 15.735 |
| 82354 | 82842 | Reverse | 162 | Tail completion or Neck1 protein | *Escherichia* phage phi92 | 99 | 3.00E-115 | 100 | YP_009012459.1 | 18.151 |
| 82842 | 83390 | Reverse | 182 | Tail capping protein | *Escherichia* phage vB_vPM_PD06 | 99 | 6.00E-120 | 100 | YP_009984719.1 | 20.139 |
| 83403 | 83753 | Reverse | 116 | Hypothetical protein | *Escherichia* phage phi92 | 99 | 3.00E-80 | 100 | YP_009012457.1 | 13.016 |
| 83839 | 84840 | Reverse | 333 | Major capsid protein | *Escherichia* phage vB_EcoM_PHB05 | 99 | 0.00E+00 | 100 | YP_009984484.1 | 37.086 |
| 84862 | 85254 | Reverse | 130 | Head decoration | *Escherichia* phage vB_EcoM_PHB05 | 99 | 8.00E-87 | 100 | YP_009984483.1 | 13.841 |
| 85273 | 86388 | Reverse | 371 | Putative scaffold protein | *Escherichia* phage vB_vPM_PD06 | 96 | 0.00E+00 | 99.44 | YP_009984723.1 | 41.014 |
| 86391 | 86870 | Reverse | 159 | DNA methyltransferase | *Escherichia* phage phi92 | 99 | 7.00E-114 | 100 | YP_009012453.1 | 18.217 |
| 86967 | 88535 | Reverse | 522 | Portal protein | *Escherichia* phage phi92 | 99 | 0.00E+00 | 99.81 | YP_009012452.1 | 58.019 |
| 88633 | 90717 | Reverse | 694 | Terminase large subunit | *Escherichia* phage phi92 | 99 | 0.00E+00 | 99.86 | YP_009012451.1 | 79.201 |
| 90726 | 91010 | Reverse | 94 | o-spanin | *Escherichia* phage EmilieFrey | 98 | 6.00E-61 | 100 | QXV78131.1 | 10.307 |
| 90961 | 91377 | Reverse | 138 | i-spanin | *Escherichia* phage EC_OE_11 | 99 | 2.00E-93 | 97.1 | WBF54129.1 | 15.650 |
| 91428 | 91601 | Reverse | 57 | Hypothetical protein | *Escherichia* phage phi92 | 98 | 4.00E-33 | 100 | YP_009012448.1 | 6.948 |
| 91740 | 92030 | Reverse | 96 | Hypothetical protein | *Escherichia* phage phi92 | 98 | 2.00E-62 | 100 | YP_009012447.1 | 11.037 |
| 92137 | 92406 | Reverse | 89 | Hypothetical protein | *Escherichia* phage phi92 | 98 | 6.00E-59 | 100 | YP_009012446.1 | 10.562 |
| 92518 | 92593 | Reverse |  | Ile |  |  |  |  |  |  |
| 92740 | 92818 | Reverse |  | Met |  |  |  |  |  |  |
| 92834 | 92909 | Reverse |  | Met |  |  |  |  |  |  |
| 92998 | 93082 | Reverse |  | Leu |  |  |  |  |  |  |
| 93159 | 93233 | Reverse |  | Phe |  |  |  |  |  |  |
| 93239 | 93314 | Reverse |  | Pro |  |  |  |  |  |  |
| 93322 | 93396 | Reverse |  | Gln |  |  |  |  |  |  |
| 93496 | 93570 | Reverse |  | Gly |  |  |  |  |  |  |
| 93626 | 93949 | Reverse | 107 | Hypothetical protein | *Escherichia* phage phiWec187 | 99 | 4.00E-70 | 98.13 | BDU12993.1 | 12747 |
| 93973 | 94047 | Reverse |  | Thr |  |  |  |  |  |  |
| 94138 | 94222 | Reverse |  | Asn |  |  |  |  |  |  |
| 94230 | 94316 | Reverse |  | Tyr |  |  |  |  |  |  |
| 94322 | 94397 | Reverse |  | Lys |  |  |  |  |  |  |
| 94523 | 94608 | Reverse |  | Ser |  |  |  |  |  |  |
| 94617 | 94769 | Reverse | 50 | Hypothetical protein | *Escherichia* phage vB_EcoM_PHB05 | 98 | 9.00E-27 | 98 | YP_009984471.1 | 5.699 |
| 94788 | 94877 | Reverse |  | Ser |  |  |  |  |  |  |
| 95203 | 95288 | Reverse |  | Leu |  |  |  |  |  |  |
| 95320 | 95395 | Reverse |  | Met |  |  |  |  |  |  |
| 95403 | 95669 | Reverse | 88 | Hypothetical protein | *Escherichia* phage vB_EcoM_PHB05 | 98 | 1.00E-54 | 100 | YP_009984470.1 | 9.794 |
| 96143 | 97876 | Reverse | 577 | Nicotinamide phosphoribosyltransferase | *Escherichia* phage vB_vPM_PD114 | 99 | 0.00E+00 | 99.83 | AXY81467.1 | 65.050 |
| 97948 | 98841 | Reverse | 297 | Ribose-phosphate pyrophosphokinase | *Escherichia* phage alia | 99 | 0.00E+00 | 99.66 | YP_009985334.1 | 33.576 |
| 99252 | 99620 | Forward | 122 | Hypothetical protein | *Escherichia* phage phi92 | 99 | 2.00E-84 | 99.18 | YP_009012437.1 | 14.118 |
| 99687 | 99884 | Forward | 65 | Hypothetical protein | *Escherichia* phage phi92 | 98 | 2.00E-39 | 100 | YP_009012436.1 | 7.625 |
| 99881 | 100120 | Forward | 79 | Hypothetical protein | *Escherichia* phage phi92 | 98 | 2.00E-51 | 100 | YP_009012435.1 | 9.154 |
| 100074 | 100409 | Forward | 111 | Transposase-like protein | *Escherichia* phage phi92 | 99 | 3.00E-72 | 100 | YP_009012434.1 | 12.649 |
| 100406 | 100684 | Forward | 92 | Hypothetical protein | *Escherichia* phage phi92 | 98 | 6.00E-60 | 100 | YP_009012433.1 | 10.505 |
| 100684 | 100875 | Forward | 63 | Hypothetical protein | *Serratia* phage KKP_3263 | 98 | 2.00E-36 | 100 | UES35815.1 | 7.239 |
| 100878 | 101219 | Forward | 113 | Hypothetical protein | *Escherichia* phage alia | 99 | 2.00E-75 | 98.23 | YP_009985327.1 | 13.135 |
| 101229 | 101378 | Forward | 49 | Hypothetical protein | *Escherichia* phage vB_vPM_PD114 | 98 | 9.00E-27 | 97.96 | AXY81458.1 | 5.957 |
| 101378 | 101737 | Forward | 119 | Hypothetical protein | *Escherichia* phage inny | 99 | 3.00E-83 | 100 | QHR69742.1 | 13.723 |
| 101737 | 102288 | Forward | 183 | phosphoesterase | *Escherichia* phage outra | 99 | 9.00E-134 | 100 | QHR75342.1 | 21.428 |
| 102290 | 103222 | Forward | 310 | RNA ligase | *Escherichia* phage muut | 99 | 0.00E+00 | 99.35 | YP_009985640.1 | 35.604 |
| 103223 | 103627 | Forward | 134 | ATPase | *Salmonella* phage UAB_1 | 99 | 5.00E-94 | 100 | UIS31464.1 | 15.680 |
| 103637 | 103957 | Forward | 106 | Hol-like chemotaxis | *Escherichia* phage phi92 | 99 | 1.00E-66 | 100 | YP_009012425.1 | 12.196 |
| 103961 | 104185 | Forward | 74 | Hypothetical protein | *Escherichia* phage phi92 | 98 | 2.00E-44 | 100 | YP_009012424.1 | 8.564 |
| 104194 | 104382 | Forward | 62 | Hypothetical protein | *Escherichia* phage phi92 | 98 | 7.00E-38 | 100 | YP_009012423.1 | 7.084 |
| 104369 | 105484 | Forward | 371 | ATP-dependent DNA ligase | *Escherichia* phage JLBYU50 | 99 | 0.00E+00 | 99.19 | UGL62203.1 | 42.156 |
| 105682 | 106404 | Forward | 240 | Nucleotide pyrophosphohydrolase | *Escherichia* phage phi92 | 99 | 1.00E-174 | 100 | YP_009012420.1 | 27.374 |
| 106441 | 106719 | Forward | 92 | Hypothetical protein | *Escherichia* phage vB_EcoM_PHB05 | 98 | 5.00E-60 | 100 | YP_009984450.1 | 10.646 |
| 106730 | 107614 | Forward | 294 | Exonuclease recombination-associated | *Escherichia* phage phi92 | 99 | 0.00E+00 | 100 | YP_009012418.1 | 33.141 |
| 107689 | 107901 | Forward | 70 | Hypothetical protein | *Escherichia* phage phi92 | 98 | 2.00E-42 | 100 | YP_009012417.1 | 7.762 |
| 107994 | 109028 | Forward | 344 | Exonuclease | *Escherichia* phage vB_EcoM_PHB05 | 99 | 0.00E+00 | 99.42 | YP_009984447.1 | 39.811 |
| 109025 | 109987 | Forward | 320 | DNA polymerase exonuclease subunit | *Escherichia* phage vB_EcoM_PHB05 | 99 | 0.00E+00 | 99.69 | YP_009984446.1 | 36.356 |
| 110026 | 110427 | Forward | 133 | Hypothetical protein | *Escherichia* phage vB_EcoM_PHB05 | 99 | 4.00E-92 | 100 | YP_009984445.1 | 15.362 |
| 110411 | 110605 | Forward | 64 | Hypothetical protein | *Escherichia* phage phi92 | 98 | 5.00E-39 | 100 | YP_009012413.1 | 7.292 |
| 110595 | 111170 | Forward | 191 | HNH endonuclease | *Escherichia* phage vB_EcoM_PHB05 | 99 | 1.00E-137 | 98.43 | YP_009984443.1 | 22.032 |
| 111216 | 111779 | Forward | 187 | Thymidine kinase | *Escherichia* phage phi92 | 99 | 5.00E-136 | 100 | YP_009012411.1 | 21.239 |
| 111763 | 112353 | Forward | 196 | Hypothetical protein | *Escherichia* phage vB_EcoM_PHB05 | 99 | 1.00E-131 | 99.49 | YP_009984441.1 | 22.704 |
| 112363 | 113334 | Forward | 323 | Thymidylate synthase | *Escherichia* phage JohannJBalmer | 99 | 0.00E+00 | 99.38 | QXV81565.1 | 37.012 |
| 113432 | 113842 | Forward | 136 | Hypothetical protein | *Escherichia* phage phi92 | 99 | 2.00E-84 | 100 | YP_009012408.1 | 15.673 |
| 113855 | 114103 | Forward | 82 | Hypothetical protein | *Escherichia* phage phi92 | 98 | 9.00E-54 | 100 | YP_009012407.1 | 9.373 |
| 114115 | 116376 | Forward | 753 | Class I ribonucleotide reductase alpha subunit | *Escherichia* phage EC_OE_11 | 99 | 0.00E+00 | 99.87 | WBF54087.1 | 85.911 |
| 116417 | 116752 | Forward | 111 | Hypothetical protein | *Escherichia* phage EmilieFrey | 99 | 6.00E-78 | 100 | QXV78350.1 | 12.721 |
| 116739 | 117824 | Forward | 361 | Class I ribonucleoside diphosphate reductase small subunit | *Escherichia* phage phi92 | 99 | 0.00E+00 | 100 | YP_009012404.1 | 41.686 |
| 117824 | 118042 | Forward | 72 | Hypothetical protein | *Escherichia* phage phi92 | 98 | 7.00E-44 | 100 | YP_009012403.1 | 7.996 |
| 118035 | 118217 | Forward | 60 | Hypothetical protein | *Escherichia* phage phi92 | 98 | 9.00E-33 | 100 | YP_009012402.1 | 6.794 |
| 118220 | 118366 | Forward | 48 | Hypothetical protein | *Escherichia* phage vB_vPM_PD06 | 97 | 2.00E-19 | 100 | YP_009984773.1 | 5.226 |
| 118356 | 118682 | Forward | 108 | Hypothetical protein | *Escherichia* phage EC_OE_11 | 99 | 1.00E-71 | 99.07 | WBF54081.1 | 12.355 |
| 118696 | 118893 | Forward | 65 | Hypothetical protein | *Enterobacteria* phage ECGD1 | 98 | 2.00E-39 | 100 | AMM43405.1 | 7.608 |
| 118890 | 119879 | Forward | 329 | Nucleotide-sugar epimerase | *Escherichia* phage vB_vPM_PD06 | 99 | 0.00E+00 | 100 | YP_009984776.1 | 37.343 |
| 119888 | 120757 | Forward | 289 | Glucose-1-phosphate thymidylyltransferase | *Escherichia* phage vB_EcoM_ESCO32 | 99 | 0.00E+00 | 100 | UPW38504.1 | 31.961 |
| 120770 | 121324 | Forward | 184 | dTDP-4-dehydrorhamnose 3.5-epimerase | *Escherichia* phage vB_EcoM_PHB05 | 99 | 5.00E-133 | 100 | YP_009984427.1 | 21.236 |
| 121321 | 122151 | Forward | 276 | dTDP-4-dehydrorhamnose reductase | *Escherichia* phage vB_vPM_PD114 | 99 | 0.00E+00 | 99.64 | AXY81424.1 | 30.267 |
| 122154 | 122414 | Forward | 86 | Thioredoxin domain | *Escherichia* phage vB_EcoM_PHB05 | 98 | 1.00E-56 | 100 | YP_009984425.1 | 9.998 |
| 122419 | 122562 | Forward | 47 | Hypothetical protein | *Escherichia* phage phi92 | 97 | 2.00E-24 | 100 | YP_009012393.1 | 5.482 |
| 122575 | 123075 | Forward | 166 | Baseplate hub subunit and tail lysozyme | *Escherichia* phage EC_OE_11 | 99 | 2.00E-117 | 99.4 | WBF54073.1 | 18.879 |
| 123114 | 123866 | Forward | 250 | PhoH-like phosphate starvation-inducible | *Escherichia* phage phi92 | 99 | 0.00E+00 | 100 | YP_009012391.1 | 27.865 |
| 123903 | 124397 | Forward | 164 | Hypothetical protein | *Escherichia* phage phi92 | 99 | 2.00E-115 | 100 | YP_009012390.1 | 19.129 |
| 124394 | 125047 | Forward | 217 | Peptidase HslV family | *Escherichia* phage muut | 99 | 2.00E-128 | 99.54 | YP_009985679.1 | 23.777 |
| 125089 | 125517 | Forward | 142 | Hypothetical protein | *Escherichia* phage phi92 | 99 | 6.00E-99 | 100 | YP_009012388.1 | 16.796 |
| 125507 | 126016 | Forward | 169 | DNA methyltransferase | *Escherichia* phage phi92 | 99 | 4.00E-109 | 98.82 | YP_009012387.1 | 19.614 |
| 126030 | 126260 | Forward | 76 | Hypothetical protein | *Escherichia* phage phi92 | 98 | 4.00E-47 | 100 | YP_009012386.1 | 8.528 |
| 126260 | 126463 | Forward | 67 | Hypothetical protein | *Escherichia* phage alia | 98 | 4.00E-40 | 100 | YP_009985282.1 | 7.518 |
| 126521 | 127183 | Forward | 220 | DNA invertase | *Escherichia* phage phi92 | 99 | 8.00E-160 | 100 | YP_009012384.1 | 24.683 |
| 127193 | 127396 | Forward | 67 | Hypothetical protein | *Escherichia* phage phi92 | 98 | 3.00E-40 | 100 | YP_009012383.1 | 7.690 |
| 127408 | 127980 | Forward | 190 | Histidyl tRNA synthetase | *Escherichia* phage phi92 | 99 | 2.00E-135 | 99.47 | YP_009012382.1 | 20.878 |
| 127991 | 128317 | Forward | 108 | Hypothetical protein | *Escherichia* phage phi92 | 99 | 3.00E-71 | 100 | YP_009012381.1 | 12.590 |
| 128317 | 129060 | Forward | 247 | NinI-like serine-threonine phosphatase | *Escherichia* phage JohannJBalmer | 99 | 0.00E+00 | 99.6 | QXV81536.1 | 28.173 |
| 129060 | 129380 | Forward | 106 | Hypothetical protein | *Escherichia* phage vB_EcoM_ESCO8 | 82 | 2.00E-50 | 94.32 | UPW37317.1 | 12.442 |
| 129391 | 129969 | Forward | 192 | DprA-like DNA recombination-mediator protein | *Escherichia* phage alia | 99 | 4.00E-141 | 100 | YP_009985275.1 | 21.687 |
| 129956 | 130474 | Forward | 172 | Hypothetical protein | *Escherichia* phage outra | 99 | 2.00E-121 | 98.84 | QHR75536.1 | 19.654 |
| 130471 | 130728 | Forward | 85 | Hypothetical protein | *Escherichia* phage phi92 | 98 | 3.00E-53 | 100 | YP_009012376.1 | 9.651 |
| 130814 | 131227 | Forward | 137 | Hypothetical protein | *Escherichia* phage PaulScherrer | 99 | 7.00E-95 | 100 | QXV83925.1 | 16.009 |
| 131227 | 132216 | Forward | 329 | DNA (cytosine-5-)-methyltransferase | *Escherichia* phage arall | 99 | 0.00E+00 | 99.7 | QHR67598.1 | 37.830 |
| 132216 | 132338 | Forward | 40 | Hypothetical protein | *Escherichia* phage VEcB | 97 | 7.00E-18 | 97.5 | YP_009987248.1 | 4.940 |
| 132340 | 132948 | Forward | 202 | Hypothetical protein | *Escherichia* phage vB_EcoM_ESCO8 | 99 | 3.00E-148 | 99.5 | UPW37323.1 | 23.682 |
| 133003 | 133536 | Forward | 177 | Putative aminoacyl-tRNA synthetase | *Escherichia* phage muut | 99 | 5.00E-124 | 98.87 | YP_009985696.1 | 20.403 |
| 133533 | 134273 | Forward | 246 | Hypothetical protein | *Serratia* phage KKP_3263 | 99 | 9.00E-158 | 99.59 | UES35992.1 | 28.718 |
| 134263 | 135078 | Forward | 271 | Sir2 (NAD-dependent deacetylase) | *Serratia* phage KKP_3263 | 99 | 0.00E+00 | 98.89 | UES35991.1 | 30.866 |
| 135123 | 135380 | Forward | 85 | Hypothetical protein | *Escherichia* phage vB_EcoM_PHB05 | 98 | 6.00E-57 | 100 | YP_009984402.1 | 9.587 |
| 135556 | 135777 | Reverse | 73 | Hypothetical protein | *Escherichia* phage phi92 | 98 | 2.00E-45 | 100 | YP_009012367.1 | 8.428 |
| 135862 | 136185 | Reverse | 107 | Hypothetical protein | *Escherichia* phage vB_EcoM_PHB05 | 99 | 5.00E-70 | 97.2 | YP_009984400.1 | 12.245 |
| 136194 | 136481 | Reverse | 95 | Hypothetical protein | *Escherichia* phage phi92 | 98 | 1.00E-62 | 100 | YP_009012365.1 | 11.144 |
| 136491 | 136664 | Reverse | 57 | Hypothetical protein | *Escherichia* phage muut | 98 | 2.00E-33 | 100 | YP_009985703.1 | 6.618 |
| 136821 | 137420 | Reverse | 199 | Hypothetical protein | *Escherichia* phage arall | 99 | 1.00E-140 | 98.99 | QHR67588.1 | 22.171 |
| 137434 | 137706 | Reverse | 90 | Hypothetical protein | *Escherichia* phage phi92 | 98 | 3.00E-59 | 100 | YP_009012361.1 | 10.381 |
| 137708 | 137920 | Reverse | 70 | membrane protein | *Escherichia* phage muut | 98 | 4.00E-29 | 100 | YP_009985707.1 | 8.070 |
| 137922 | 138104 | Reverse | 60 | Hypothetical protein | *Escherichia* phage phi92 | 83 | 3.00E-28 | 100 | YP_009012359.1 | 7.087 |
| 138098 | 138508 | Reverse | 136 | Hypothetical protein | *Escherichia* phage vB_EcoM_ESCO9 | 99 | 1.00E-93 | 100 | UPW37547.1 | 15.634 |
| 138614 | 138862 | Reverse | 82 | Hypothetical protein | *Escherichia* phage inny | 98 | 1.00E-52 | 100 | QHR69673.1 | 9.487 |
| 138859 | 139122 | Reverse | 87 | Hypothetical protein | *Escherichia* phage arall | 98 | 5.00E-57 | 100 | QHR67582.1 | 9.753 |
| 139122 | 139340 | Reverse | 72 | Hypothetical protein | *Escherichia* phage phi92 | 98 | 1.00E-44 | 98.61 | YP_009012355.1 | 8.351 |
| 139548 | 139790 | Reverse | 80 | Hypothetical protein | *Escherichia* phage vB_EcoM_PHB05 | 98 | 1.00E-35 | 98.75 | YP_009984389.1 | 9.158 |
| 139783 | 140007 | Reverse | 74 | Hypothetical protein | *Enterobacteria* phage ECGD1 | 98 | 2.00E-23 | 98.65 | AMM43363.1 | 8.663 |
| 140061 | 140234 | Reverse | 57 | Hypothetical protein | *Escherichia* phage phi92 | 98 | 7.00E-35 | 100 | YP_009012352.1 | 6.838 |
| 140231 | 140482 | Reverse | 83 | Hypothetical protein | *Enterobacteria* phage ECGD1 | 98 | 7.00E-52 | 100 | AMM43361.1 | 9.454 |
| 140479 | 140778 | Reverse | 99 | Hypothetical protein | *Escherichia* phage muut | 99 | 6.00E-65 | 100 | YP_009985717.1 | 12.180 |
| 140778 | 141017 | Reverse | 79 | Hypothetical protein | *Escherichia* phage phi92 | 98 | 3.00E-49 | 100 | YP_009012349.1 | 8.881 |
| 141008 | 141202 | Reverse | 64 | Hypothetical protein | *Escherichia* phage vB_EcoM_PHB05 | 98 | 5.00E-40 | 100 | YP_009984384.1 | 7.324 |
| 141204 | 141662 | Reverse | 152 | Hypothetical protein | *Escherichia* phage alia | 99 | 2.00E-110 | 100 | YP_009985246.1 | 17.878 |
| 141679 | 142023 | Reverse | 114 | Hypothetical protein | *Escherichia* phage JohannJBalmer | 99 | 3.00E-55 | 100 | QXV81505.1 | 13.051 |
| 142025 | 142198 | Reverse | 57 | Hypothetical protein | *Escherichia* phage vB_EcoM_PHB05 | 94 | 9.00E-32 | 100 | YP_009984381.1 | 7.063 |
| 142198 | 142782 | Reverse | 194 | Hypothetical protein | *Escherichia* phage EC_OE_11 | 99 | 5.00E-143 | 99.48 | WBF54270.1 | 23.154 |
| 142961 | 143185 | Reverse | 74 | Hypothetical protein | *Escherichia* phage muut | 97 | 7.00E-47 | 100 | YP_009985727.1 | 8.430 |
| 143195 | 143413 | Reverse | 72 | Hypothetical protein | *Escherichia* phage phi92 | 98 | 9.00E-33 | 95.83 | YP_009012338.1 | 8.524 |
| 143406 | 143756 | Reverse | 116 | Hypothetical protein | *Escherichia* phage vB_EcoM_ESCO32 | 99 | 6.00E-82 | 100 | UPW38561.1 | 13.990 |
| 143770 | 144069 | Reverse | 99 | Hypothetical protein | *Escherichia* phage vB_vPM_PD06 | 99 | 1.00E-67 | 100 | YP_009984833.1 | 11.317 |
| 144066 | 144197 | Reverse | 43 | Hypothetical protein | *Enterobacteria* phage ECGD1 | 97 | 9.00E-21 | 100 | AMM43348.1 | 5.017 |
| 144282 | 144734 | Reverse | 150 | Hypothetical protein | *Escherichia* phage outra | 99 | 8.00E-103 | 98 | QHR75496.1 | 17.380 |
| 144750 | 144965 | Reverse | 71 | Hypothetical protein | *Escherichia* phage muut | 98 | 4.00E-33 | 98.59 | YP_009985734.1 | 8.065 |
| 144967 | 145425 | Reverse | 152 | Hypothetical protein | *Escherichia* phage EmilieFrey | 99 | 9.00E-108 | 97.37 | QXV78275.1 | 17.499 |
| 145496 | 145861 | Reverse | 121 | Hypothetical protein | *Salmonella* phage UAB_1 | 99 | 1.00E-85 | 100 | UIS31372.1 | 14.130 |
